# Degradation of Plastics under Anaerobic Conditions: A Short Review

**DOI:** 10.3390/polym12010109

**Published:** 2020-01-05

**Authors:** Xochitl Quecholac-Piña, María del Consuelo Hernández-Berriel, María del Consuelo Mañón-Salas, Rosa María Espinosa-Valdemar, Alethia Vázquez-Morillas

**Affiliations:** 1 Tecnológico Nacional de México/Instituto Tecnológico de Toluca, Av. Tecnológico s/n. Colonia Agrícola Bellavista Metepec, Edo. De México, México C.P. 52149, Mexico; xquecholacp@toluca.tecnm.mx (X.Q.-P.); mhernandezb@toluca.tecnm.mx (M.d.C.H.-B.); 2 Sociedad Mexicana de Ciencia y Tecnología Aplicada a Residuos Sólidos, A.C., Priv Molcajete 44 Fracc. Hacienda de las Fuentes, Calimaya, México C.P. 52227, Mexico; consuelomanon@gmail.com; 3 Universidad Autónoma Metropolitana, Av San Pablo Xalpa 180, Reynosa Tamaulipas, Azcapotzalco, Ciudad de México 02200, Mexico; rmev@azc.uam.mx

**Keywords:** anaerobic digestion, landfill, biogas, mineralization, biodegradation, biodegradable plastics

## Abstract

Plastic waste is an issue of global concern because of the environmental impact of its accumulation in waste management systems and ecosystems. Biodegradability was proposed as a solution to overcome this problem; however, most biodegradable plastics were designed to degrade under aerobic conditions, ideally fulfilled in a composting plant. These new plastics could arrive to anaerobic environments, purposely or frequently, because of their mismanagement at the end of their useful life. This review analyzes the behavior of biodegradable and conventional plastics under anaerobic conditions, specifically in anaerobic digestion systems and landfills. A review was performed in order to identify: (a) the environmental conditions found in anaerobic digestion processes and landfills, as well as the mechanisms for degradation in those environments; (b) the experimental methods used for the assessment of biodegradation in anaerobic conditions; and (c) the extent of the biodegradation process for different plastics. Results show a remarkable variability of the biodegradation rate depending on the type of plastic and experimental conditions, with clearly better performance in anaerobic digestion systems, where temperature, water content, and inoculum are strictly controlled. The majority of the studied plastics showed that thermophilic conditions increase degradation. It should not be assumed that plastics designed to be degraded aerobically will biodegrade under anaerobic conditions, and an exact match must be done between the specific plastics and the end of life options that they will face.

## 1. Introduction

Plastics are materials formed by polymers and additives, characterized by high molecular weight. Their versatility makes them an essential material in different applications; they are used in packaging (39.9%), construction (19.8%), the automotive industry (9.8%), electronics and communication (6.2%), agriculture (3.4%), household leisure and sport (4.1%), and other fields (16.7%) [1]. Among the different plastics, the most commonly used in packaging are polypropylene (PP), low-density polyethylene (LDPE), high-density polyethylene (HDPE), vinyl chloride (PVC), polyurethane (PU), polyethylene terephthalate (PETE), and polystyrene (PS). Most plastics are durable and resistant to degradation; this is the feature that makes them very attractive for many applications. However, these characteristics have adverse effects once plastic products are discarded. In 2015, 6300 Mt of plastic waste was produced; 9% was recycled, 12% was incinerated, and 79% was landfilled or littered into the environment [2]. In Mexico, plastics account for 11% of the urban solid waste [3] (INEGI, 2017), but only 5% of the plastic waste is recycled, while 40% goes to landfill and 32% ends in the natural environment. Improper management and accumulation of plastic waste affect the ecosystems, wildlife, and the quality of human life. Specific effects include entanglement, blockage of the digestive tract of different species, the formation of microplastics, and loss of aesthetic value [4,5].

Different solutions have been proposed to solve the problems associated with plastic waste. They include recycling, energy recovery, ban of specific products, and production of biodegradable plastics. Recycling is an attractive option for thermoplastics, especially for those used in high volumes, such as PETE and polyethylene (PE). It can diminish the use of natural resources and global warming [6,7]; however, it requires separation techniques that produce pure raw materials that can be incorporated into new products [8,9,10]; this need for segregation hinders the ability of many plastics to be recycled [11]. Energy recovery, on the other hand, can be achieved through direct combustion or by the production of refuse-derived fuel [12,13,14,15]; one of its advantages is its capacity to treat mixed plastics. However, special care is needed when dealing with chlorinated plastics, such as PVC, whose incineration can lead to the formation of acid emissions [10]. Waste to energy also requires strict control of emissions to prevent the formation of toxic compounds, such as dioxins and furanes [10,16]. The technology required in order to achieve a safe energy recovery process makes it non-viable for some developing countries, due to limitations in budget. Another approach to controlling plastic pollution has been the ban on specific products, mainly carrier bags. Regulations that restrict the use of plastic bags or tax them have been proposed in cities of countries like South Africa [17], Canada [18], the United States [19], Portugal [20], Malaysia [21], Argentina [22], Mexico [23], Japan [24], England [25], China [26], and Kenya [27], among others.

Another alternative for the control of plastic waste is the production of plastics that degrade under specific conditions, such as composting, aquatic environments, and landfills [28]. The final goal of these option is to reintegrate the elemental components of the plastics to the natural cycles, diminishing the effects related to their accumulation. Based on the factor that causes degradation, different types of degradable plastics can be distinguished [29]: (a) photodegradables, whose polymeric chains are fragmented due to the formation of free radicals produced by UV radiation; (b) oxodegradable, which contain an additive that promotes their abiotic degradation until they reach a size that can be degraded biotically; and (c) biodegradable, which can be directly degraded by living organisms, such as bacteria, fungi, and algae, to produce mineral gases and biomass. In order to take advantage of the specific features of these plastics, different conditions must be fulfilled, such as an articulated system of certification, eco-labeling and separation at source [28].

While a degradable plastic can be any plastic designed to undergo a significant change in its chemical structure under specific environmental conditions, biodegradable plastics require that change be caused explicitly by the action of naturally-occurring micro-organisms [29]. In principle, any organic compound is biodegradable [30]. However, as the biodegradation process of plastics is expected to occur along with the management of urban solid waste, different factors need to be taken into account. The first one is the specific environment in which biodegradation would take place, which could include any valid end-of-life option with a high concentration of microorganisms, such as composting, anaerobic digestion processes, or landfills. Even if natural environments could provide conditions that lead to biodegradation, they should not be considered as an acceptable final sink for plastics. The second factor is the speed of the process, as these plastics should degrade at a similar rate than other knowingly biodegradable substrates, like food and yard waste [31,32]. The third factor that must be considered is the extent of biodegradation, which should lead to mineralization, allowing it to close the carbon biogeochemical cycle [30]. If these factors are adequately addressed, the carbon-content of biodegradable plastics would be converted, in a reasonable time-frame, in new biomass and CO_2_. This result clearly differentiates biodegradation from oxidative and UV-promoted degradation processes, which could lead to the formation of microplastics (<5 mm) [33,34]. Microplastics pose a severe risk to marine ecosystems, as they can enter the food chain when they are ingested by different species [35,36], adsorb hydrophobic pollutants [37,38], and leach additives [39,40]. Besides their presence in the ocean, they have been found in freshwaters [41,42], soils [43,44], and in the atmosphere [45,46], giving evidence of their ubiquitous presence in the environment.

The majority of biodegradable plastics have been designed to degrade in the aerobic environment of an industrial composting plant [47]. However, in developing countries, they can be disposed in landfills due to deficient management practices, misinformation, and lack of infrastructure. Also, they could be incorporated in anaerobic digestion systems in places where this technology is used to treat organic waste. While anaerobic digestion systems are specifically designed to promote the biodegradation process through the control of operational variables, the main objective in landfills is the safe confinement of waste, even if in some of them the produced biogas is collected and used [48]. Anaerobic digestion processes are usually applied to organic waste with a low content of inorganics that is processed in a defined time-frame in a closed reactor [49,50]. The process can be carried on at mesophilic or thermophilic temperatures, and inoculum is added in order to increase the rate and extent of biodegradation; optimization of solid content, carbon/nitrogen ratio and food/inoculum ratio is carried on to increase methane production [51]. In landfills, on the other hand, a complex and heterogeneous mixture of waste is buried, and the degradation process occurs as a result of the activity of bacteria present naturally in the waste. The inherent heterogeneity of waste, as well as the different environmental and operational conditions, will produce an extensive range of biodegradation processes that usually will extend through decades. In landfills, the rate of biodegradation increases as leachate is recirculated [52,53,54]. However, they are slower than in anaerobic digestion systems.

In this context, this review aims to offer an assessment of the behavior of different biodegradable plastics under anaerobic conditions that would take place in anaerobic digestion systems and landfills.

The inclusion of these new plastics in engineered anaerobic systems could contribute to methane production. In this context, this review includes a description of the anaerobic biodegradation of plastics that takes place in anaerobic digestion processes and landfills, in order to explain the mechanisms that take place in the environments mentioned above. An analysis of the different experimental conditions used in the assessment of this kind of systems is presented in order to identify common features. Following this, a summary and analysis of the results obtained for different biodegradable and conventional plastics are shown. Natural anaerobic environments are not included in this research, as they must not be considered as viable waste management options.

## 2. Methods

The search for research articles and book chapters was done in the first semester of 2019, in the scientific finders Scopus^®^, Access DL^®^, Oxford Academy^®^, Springer link^®,^ and Science Direct^®^. The initial keywords used were plastics plus anaerobic, degradation, polymers, and landfill. A total of 1207 abstracts were read to identify papers focused on the degradation of plastics under anaerobic conditions. This process allowed us to obtain 70 articles from 34 different peer-reviewed journals (see reference material for the complete list of articles). References were downloaded in format bibTex in the free software Mendeley^®^.

The scientific articles and chapters were analyzed in order to identify information related to the following topics: (a) scenarios and mechanisms for anaerobic degradation of plastics, (b) methods for the assessment of the anaerobic degradation of plastics, and (c) degradation of plastics under anaerobic digestion and landfill conditions.

## 3. Results

The different research papers used in this review, as well as their characteristics and main results are presented in the Supplementary Materials.

### 3.1. Biodegradable Plastics Assessed under Anaerobic Conditions

Biodegradable plastics include polylactic acid (PLA), polycaprolactone (PCL), polybutylene succinate (PBS), poly(butyleneadipate-coterephtalazte) (PBAT), poly(hydroxybutyrate) (PHB) and poly(hydroxybutyrate)-co-poly(hydroxyvalerate) (PHBV). Except for PBAT, they are aliphatic polyesters. They can be produced from biomass or fossil fuels. Table 1 shows the monomers of the most commonly biodegradable plastics assessed under anaerobic conditions.

#### 3.1.1. Anaerobic Processes in Digestion Systems and Landfills

Anaerobic degradation is a biological process that transforms organic matter in an oxygen-free environment. It can follow two routes: Anaerobic fermentation, where organic matter can act as an electron donor or receptor; or anaerobic respiration, which requires acceptors such as CO_2_, SO_4_^2−^, NO_3_^−^. The process is developed in four stages: Hydrolysis, acidogenesis, acetogenesis, and methanogenesis, which lead to the production of a mixture of CH_4_ and CO_2_, known as biogas. Two thirds of CH_4_ produced in an anaerobic process is because of the fermentation and one third by respiration [58]. The efficiency of the anaerobic biodegradation is affected by the presence of volatile fatty acids, sulfate, ammonia, and heavy metals [59], and for factors such as pH, temperature [58], redox potential, and hydrogen concentration [58,60]. It will also be dependent on the concentration and type of microorganisms present in the media [61,62], presence of nutrients [58], and the characteristics of the substrate [63,64,65].

In landfills, the inherent heterogeneity of waste increases the complexity of the biodegradation process [66]. Anaerobic biodegradation in these sites is developed in five stages [67]. In the first one hydrolysis takes place, producing a decrease in pH, an increase in the chemical oxygen demand and concentration of volatile fatty acids, ammonia and sulfates. In the second stage, acidogenesis produces H_2_. In the third stage, acetogenic fermentation leads acetic acid, hydrogen, and CO_2_ being formed. In the fourth stage, methanogenesis leads to biogas production. In the last stage, stabilization shows a continuous decrease in the generation of biogas, increasing the concentration of N_2_. The biodegradation process is affected by temperature, oxygen, moisture content, alkalinity, presence of nutrients, and inhibitors [68]. Although it is not feasible to control all these parameters in a landfill, it has been proved that the treatment and recirculation of leachate can increase the biodegradation rates, promoting the functioning of the landfill as a bioreactor [68].

#### 3.1.2. Degradation Mechanisms

The degradation of plastics is usually a concurrent phenomenon involving the interaction of physical, chemical, and biological factors. The resulting mechanism will depend on the nature of the material and the environmental conditions [69]. The factors that produce different degradation processes have been described previously [69,70,71]; they include UV radiation, temperature, mechanical stress, oxidative, hydrolysis, and biodegradation processes. Oxidation by oxygen and ozone will not be relevant in the case of anaerobic degradation of plastics, and the effect of photo-oxidation will be limited, as anaerobic systems are commonly carried on in closed vessels or confined spaces [72,73,74].

For plastics whose structure includes a carbon backbone, such as polyolefins and polystyrene [75,76], mechanisms based in the formation of free radicals that promote consecutive cleavages of the polymeric chain have been proposed; reactions begin in tertiary carbons. For polyethylene (PE), it has been proposed a mechanism for UV-promoted radiation, which produces CH_4_ and H_2_ as a result of the formation of free radicals [75].

On the other hand, polymers containing elements different from carbon in their main chain can undergo hydrolysis processes. The first step is the diffusion of water through the material, especially in amorphous regions that present lesser resistance. The water promotes hydrolytic rupture of the steric bonds, which causes a decrease in molecular weight and formation of water-soluble byproducts. These reactions decrease the stability of the crystalline zones of the plastic because of an autocatalytic process promoted by the degradation of acid products that increase the presence of carboxylic acids in the extremes of the polymers [77]. This type of mechanism has been proposed for the degradation of polycaprolactone (PCL) and polylactic acid (PLA). The rate of degradation increases as the reaction proceeds, and it is affected by temperature and extreme pH values [77].

The mechanisms proposed for the biodegradation of plastics such as PHBV begin with the colonization of the material surface, followed by hydrolysis, erosion of the exposed areas, and saturation that leads to fragmentation [78]. A similar sequence has been described for copolymers of polyethylene and starch, where the microorganisms attack the amorphous starch zones in the surface, allowing their entrance to internal pores [79]. However, once the starch has been consumed, the degradation of the polyethylene matrix can be obtained by chemical factors.

### 3.2. Assessment of the Biodegradation Process under Anaerobic Conditions

**Figure 1:**
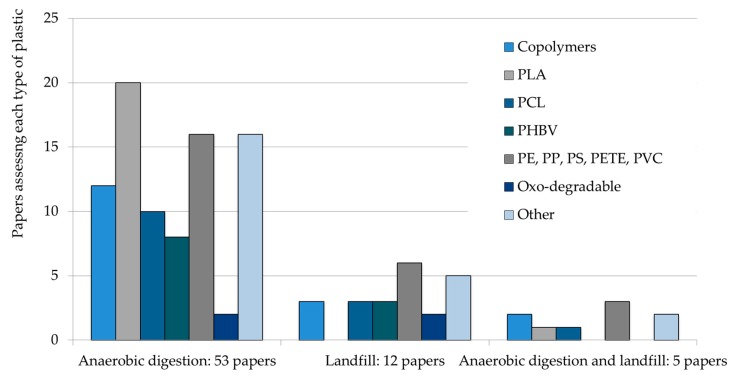
Biodegradation of different plastics under anaerobic conditions. Abbreviations: PLA, polylactic acid; PCL, polycaprolactone; PHBV, poly(hydroxybutyrate)-co-poly(hydroxyvalerate); PE, polyethylene; PP, polypropylene; PS, polystyrene; PETE, polyethylene terephthalate; PVC, vinyl chloride.

The study of the anaerobic degradation of biodegradable plastics has focused on PLA, PCL, PHBV, and copolymers that include two different biodegradable plastics, or a conventional plastic mixed with a biodegradable one. Conventional plastics (PE, PETE, PP, PS, PVC) have also been tested in order to measure their degradation, their possible adverse effects in the biodegradation processes, or in the case of polyethylene, as negative controls for the experiments. The distribution of tested plastics for anaerobic digestion and landfill conditions is shown in Figure 1. While 76% of the reviewed studies have tested degradation under anaerobic digestion conditions, 17% have tried to simulate landfill conditions and 7% tested both scenarios.

#### 3.2.1. Experimental Setup

**Figure 2:**
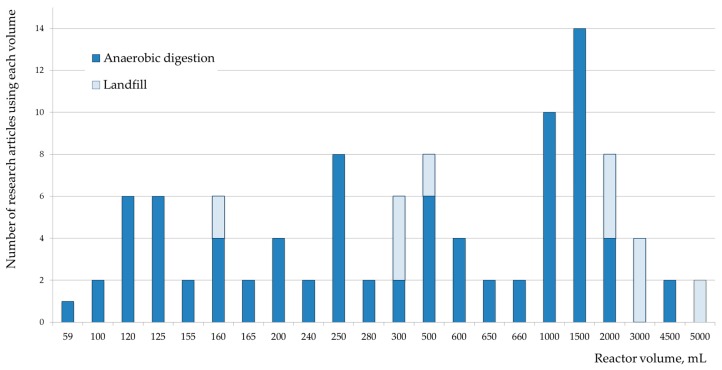
Volume of bioreactors used in the assessment of plastics.

The assessment of the anaerobic biodegradation of plastics is usually done at lab scale, because of economic and logistic reasons that prevent the development of field studies; only nine studies tested full-scale conditions, exposing the plastics in landfill cells. In laboratory experiments, the volume of the used bioreactors varies from 0.05 L to 5.0 L, as shown in Figure 2. While 28% of the experiments were performed in small reactors (0.1–0.2 L), 31% were developed in medium-size reactors (1.0–2.0 L) and only 8% were done in reactors bigger than 2.0 L. There is no a discernible pattern for the volume of reactors in the assessment of anaerobic digestion and sanitary landfill; small and big reactors were used to simulate both conditions. Very likely, the selection of the reactor’s size was related to the availability of resources, and not to specific goals or designs.

The length of the experiments is related mainly to the type of scenario that is being evaluated. Anaerobic digestion is a relatively fast process, which usually lasts 15 to 45 days. This condition is easily replicated in the laboratory, and is commonly used to test the inherent anaerobic biodegradability of new materials. Fifty-two percent of the reviewed papers focused on anaerobic digestion that lasted from 7 days to three months. On the other hand, the biodegradation process taking place in a landfill will extend for a much longer timeframe, which can take up to 20 years. To simulate those conditions, some of the experiments extended up to six years; in fact, the four longest studies (2–6 years) are simulations of landfill conditions. In order to increase the biodegradation rates of reactors simulating landfills, leachate can be recirculated, as has been done in different studies [80,81,82].

The presence of a mixture of microorganisms capable of degrading waste is a necessary condition in order to achieve an efficient process. To guarantee this condition, the microbial population can be increased by the introduction of an inoculum [65]. To increase the quality and production of biogas, it is convenient to pre-incubate the microorganisms until 60% of CH_4_ is reached [83]. This enrichment is commonly done in anaerobic digestion tests [61,62,63,83,84,85,86,87].

#### 3.2.2. Analytical Techniques Used to Assess Plastic Degradation and Biodegradation

Different analytical techniques have been used in order to identify or measure the degradation of plastics in anaerobic environments. The main quantitative method is respirometry, which measures the cumulative biogas or methane production in reactors containing the assessed polymer, comparing it with controls that do not contain the tested material [87]. Respirometric tests allow the quantification of biodegradation, which is expressed as a percentage. Usually, the CH_4_ or biogas produced is corrected subtracting the amount produced in a blank and then is compared to the theoretical production, based on the carbon content in the sample [84,88,89,90,91,92,93,94]. Variations of this method include the measurement of CO_2_, and the inclusion of liquid and gas phases [87,95]. Modified Gompertz equations, which considers a latency factor, were used [50,60,61] to model the biodegradation process.

The international associations ASTM International and ISO have defined standards for the assessment of biodegradation of plastics under anaerobic conditions, based on respirometric measurements [96,97,98,99,100,101] (Table 2).

**Figure 3:**
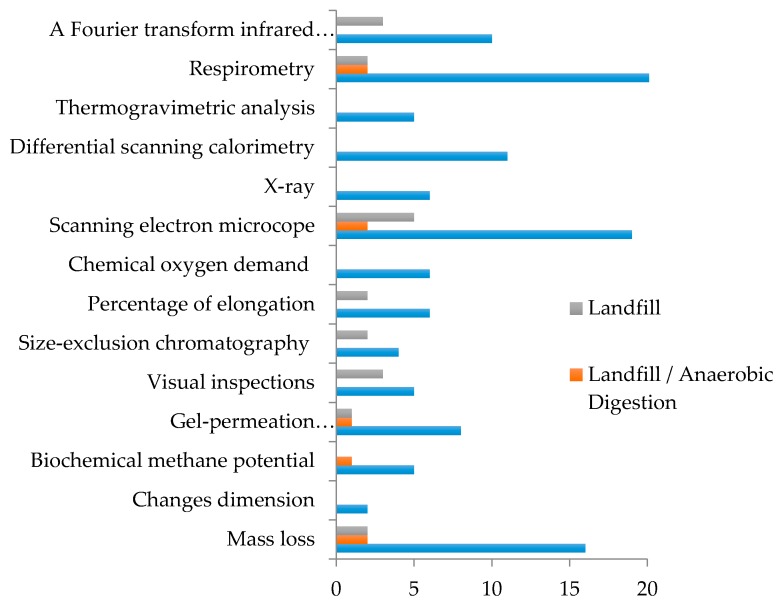
Frequency of methods used to assess plastic degradation and biodegradation.

Respirometric systems rely on the use of closed reactors, which allow the collection of all the gases produced; such conditions are difficult to obtain in big size reactors or field trials. In those cases, the assessment of the process is usually done by the direct analysis of the physical or chemical properties of the polymers [102]. The analyzed properties include loss of mass, loss of mechanical properties, changes in the thermal behavior of the materials, microbial colonization of the surface observed by scanning electron microscopy, presence of specific functional groups measured by infrared spectroscopy, or decrease in molecular weight, assessed by gel permeation or exclusion chromatography, among others [86,87,91,103,104,105,106,107]. While these techniques can give evidence of degradation, they do not allow for the obtaining od a quantitative measurement of degradation or to distinguish between degradation caused by microorganisms and other types of degradation processes. The frequency of use of these techniques is shown in Figure 3.

### 3.3. Degradation of Different Plastics in Anaerobic Environments

#### 3.3.1. Anaerobic Digestion

Biodegradable plastics have shown different biodegradation rates, depending on experimental conditions, as shown in Table 3. Studies comparing different polyhidroxialcaonates reported a 62% conversion to biogas [108,109], and an enhancement in biogas production when this plastic was present. In the specific case of PHB, it degraded 90% in 14 days at 55 °C, with a rate similar to cellulose [86]. However, it only reached 23% in 10 weeks when degrading with anaerobic sludge [89]. For PCL, reported biodegradation ranged from 80% in 50 days at 55 °C [86] to neglectable values [89,110], in some cases showing only changes in color [105]; the degradation increased when eggshell was used to produce a biocomposite [110]. Similar behavior was shown by PBS, whose degradation reached values from 25.8% under thermophilic conditions [83] and 32.5% [88], but did not show mineralization in experiments carried on at 55 °C [86]. Reported biodegradation for PLA varies from total mineralization [111], 75% in 75 days in thermophilic conditions [86], as well as 60% [88] and complete lack of biodegradation in 100 days of experimentation [112]. PBAT degraded 9.3% in 75 days [88]. Biodegradation rates of different plastics have been affected by the size of the plastic samples [103], the use of cosubstrates, and alkalinity [111]. One of the main factors to promote the process was temperature, showing higher rates for thermophilic rather than mesophylic conditions [108,113]. It was proposed that biodegradation increases when the vitrea transition temperature of the plastics was reached in the system [114].

Conventional plastics, such as cellulose acetate [115], have shown negligible biodegradation, as well as PETE [107]. The feasibility of biodegradation of PVC was obtained only if the plastic was subjected previously to a Fenton oxidation process [93]. In fact, inhibition of biogas production was reported due to the presence of HDPE, PP, and PS [116]. The addition of additives that promote abiotic oxidation to transform them into oxodegradable plastics did not promote biodegradation of PE and PETE [106]. On the other hand, PETE-PLA copolymers reached 34–69% mineralization, depending on the starting aromatic to aliphatic ratio [107]. Similarly, the use of starch in composites with PE only promoted biodegradation of the bio-based polymer [102].

In recent years there was increasing interest in the composites containing bio-based dispersed phases, known as biocomposites. Inclusion of bio-based phases was expected to decrease the environmental impact of conventional plastics because of their renewable origin and in some cases, biodegradability [117]. Starch was selected for many biocomposites, given its low cost, good permeability properties, and inherent biodegradability by microorganisms into harmless products [118]. However, evidence shows that these composites are not inherently biodegradable; for composites based in polyolefins, biodegradation occurs only for starch [119]. It was found that the starch in these composites biodegrades only partially, as it is limited if a discontinuous matrix of starch is present [120]. For composites based in a biodegradable matrix, different biodegradation rates ranging from 10.2% to 53% were reported [121,122,123].

Table 3 summarizes the results for biodegradation under anaerobic digestion conditions, showing only those where respirometric tests were used to assess the process. It can be observed that PHB, PHBV, and their copolymers achieved high levels (>80%) of mineralization, while PCL and PLA results varied widely without showing a discernable pattern related to temperature or length of the experiments. On the other hand, PBS, conventional non-degradable plastics, and their combination with pro-oxidant additives did not achieve significant biodegradation.

#### 3.3.2. Landfill

PLA did not biodegrade in simulated landfill conditions, with different recirculation rates [68], due to the anaerobic conditions that decrease the rate of the initial hydrolysis step. However, a 90% mass loss was reported when thermophilic conditions were reached [59]. Degradation in landfill conditions was influenced by the presence of oxygen in the media; while PHBV degraded almost 100% in simulated conditions with forced aeration, under strictly anaerobic conditions degradation was negligible [42]. The effect can be related to the higher concentration of microorganisms found in the higher layers of waste cells, as shown in the study of Menmee et al. [129].

Conventional, not biodegradable plastics, have shown meager degradation rates in simulated and real landfill conditions. This was found for PU [135], cellulose acetate [65], PP [136], and PVC [137]. Polyolefinic plastics (HDPE, LDPE, PP) and PS degraded between 4.96% and 15% in lysimeters that simulated landfill conditions [129]. PE and PETE with biodegradation-promoting additives showed no biodegradation under soil burial [106] when the presence of oxygen was similar to the one that can be found in a landfill.

## 4. Conclusions

Biodegradable plastics were presented as a solution to the accumulation of these materials in landfills and natural ecosystems. While most of them were designed to degrade in aerobic composting systems, two scenarios could lead to their presence in anaerobic processes. The first being the interest in degrading them in digestion systems used to degrade organic waste and produce biogas. The second being that their presence in landfills and dumpsites due to mismanagement or lack of infrastructure. This latter situation is very likely to happen in developing countries, where segregation and separate treatment of different fractions of waste is scarce.

As expected, biodegradation of plastics is higher in digestion systems than in simulated landfill conditions because of the control of temperature, agitation, mixing, solids concentration, and presence of microorganisms, among other factors that can be regulated in digestion. Biodegradable plastics showed higher mineralization than conventional ones in digestion and landfill experiments. However, this research showed clearly that experimental conditions could lead to different and even opposite results, varying parameters such as time of residence, recirculation of leachate, and aeration. Besides, the use of biocomposites or copolymers increases the variability of results.

Even if standard ISO and ASTM methods are defined for the assessment of these materials, there are not current specifications that define mineralization rates that a plastic should reach in order to be labeled as “biodegradable in anaerobic digestion” or “biodegradable in landfill”. This situation makes a comparison between materials difficult and prevents a complete understanding of how they will react in real waste management conditions.

While in developed countries, standards and ecolabels are used to asses and explain the expected behavior of materials in different waste management options, many countries do not have these essential tools. In this context, it is common for different materials to be announced as “biodegradable” without information regarding the fate that these new plastics will have in the specific waste management conditions that they will face. In developing countries, the most likely end of life scenario will be a landfill or dumpsite, where the biodegradation rate of biodegradable plastics will be slow. Even if mineralization occurs, it could lead to greenhouse emissions if not suitable systems for biogas recovery are in place.

To increase the contribution of biodegradable plastics to the solution of the waste accumulation, further research is needed in order to increase understanding of their behavior in waste management systems. This knowledge has to be shared with consumers, decision-makers, and waste management operators to promote proper management of these new materials.

## Figures and Tables

**Table 1:** Common monomers in biodegradable plastics.

Biodegradable Plastic	Acronym	Monomer	Reference
Polylactic acid	PLA	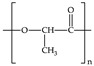	[55]
Polycaprolactone	PCL	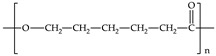	[55]
Polybutylene succinate	PBS	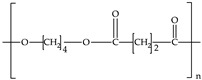	[56]
Poly(butyleneadipate-co-terephthalate)	PBAT	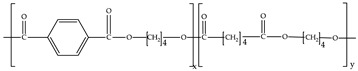	[57]
Poly-(3-hydroxyburyate)	PHB	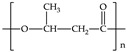	[55]
Polyhydroxybutyrate-co-polyhydroxyvalerate	PHBV	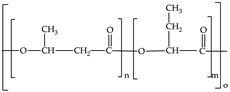	[55]

**Table 2:** Standards for the assessment of biodegradation of plastics in anaerobic environments.

Method	Description
ASTM D5511-18	Biodegradation under high-solids (>30%) anaerobic digestion conditions
ASTM D7475-11	Aerobic degradation and anaerobic biodegradation of plastic materials under accelerated bioreactor landfill conditions
ASTM D5526-18	Anaerobic biodegradation under accelerated landfill conditions
ISO 15985: 2014	Anaerobic biodegradation under high-solids anaerobic digestion conditions (solids <20%)
ISO 13975: 2019	Anaerobic biodegradation in controlled slurry digestion systems (solids <15%)
ISO 14853: 2016	Anaerobic biodegradation in aqueous systems

References: [96,97,98,99,100,101].

**Table 3:** Biodegradation of different biodegradable and conventional plastics in anaerobic digestion. Abbreviation: PBS, polybutylene succinate, PCL, polycaprolactone, PHB, poly-(3-hydroxyburyate), PHBV, PLA, polylactic acid, PVA, polyvinyl alcohol, PETE, polyethylene terephatalate, PP, polypropylene, PU, polyuretane.

Plastic	% Biodegradation	Temperature (°C)	Time (Days)	Reference
PBS	0.0	37	277	[86]
	0.0	55	50	[85]
	2.0	35	139	[124]
PCL	62.0	55	150	[125]
	12.5	37	277	[86]
	80.0	55	50	[85]
	92.0	55	75	[87]
PCL-starch	83.0	35	139	[124]
PHB	92.5	37	277	[86]
	90.0	55	50	[85]
	100	35	225	[62]
	87	35	16	[126]
PHBV	86.0	37	42	[127]
	90	35	30	[128]
	80	35	100	[112]
PHBV-PHB	96	35	16	[126]
	91.4	58	60	[119]
PLA	74.0	55	150	[119]
	39.0	37	277	[86]
	75.0	55	50	[85]
	36.0	35	170	[129]
	77.2	55	57	[129]
	70.0	55	45	[130]
	81.8	80	22	[131]
	91.5	37	100	[132]
	0.1	37	56	[132]
	98.9	58	56	[132]
	80.0	52	56	[112]
	21.0	35	75	[133]
	93.0	55	75	[133]
	79.0	55	75	[87]
	89	35	100	[112]
PLA-poly(propylene glicol)	90.0	35	182	[114]
PVA-starch	52.0	35	26	[122]
	60.0	37	115	[134]
PETE + pro-oxidant additive	2.2	37	50	[123]
PP + pro-oxidant additive	3.1	37	50	[123]
PP + starch	26.4	37	50	[123]
PU	8.95	37	105	[74]

## Supplementary Materials

The following are available online at https://www.mdpi.com/2073-4360/12/1/109/s1, Table S1: List of articles. Table S2: Summary of experimental conditions and results.

## Abbreviations

CA	cellulose acetate
HDPE	high-density polyethylene
LDPE	low-density polyethylene
PCL	polycaprolactone
PBAT	poly (butyleneadipate-co-terephthalate)
PBS	polybutylene succinate
PE	polyethylene
PETE	polyethylene terephthalate
PHA	Poly(3-hydroxyalkanoates)
PHB	poly- (3-hydroxybutyrate)
PHBH	polyhydroxybutyrate-co-polyhydroxyvalerate
PHBO	poly(3-hydroxybutyrate-*co*-3-hydroxyoctanoate)
PHBV	poly(3-hydroxybutyrate-*co*-3-hydroxyvalerate)
PLA	polylactic acid
PP	Polypropylene
PS	Polystyrene
PU	polyurethane
PVA	polyvinyl alcohol

## References

[1] PlasticsEurope (2019). Plastics-the Facts 2019 an Analysis of European Plastics Production, Demand and Waste Data.

[2] Geyer R., Jambeck J.R., Law K.L. (2017). Production, use, and fate of all plastics ever made. Sci. Adv..

[3] INEGI (2017). Anuario Estadístico y Geográfico de los Estados Unidos Mexicanos 2017.

[4] Derraik J.G. (2002). The pollution of the marine environment by plastic debris: A review. Mar. Pollut. Bull..

[5] Browne M.A., Crump P., Niven S.J., Teuten E., Tonkin A., Galloway T., Thompson R. (2011). Accumulation of Microplastic on Shorelines Woldwide: Sources and Sinks. Environ. Sci. Technol..

[6] Rajendran S., Scelsi L., Hodzic A., Soutis C., Al-Maadeed M.A. (2012). Environmental impact assessment of composites containing recycled plastics. Resour. Conserv. Recycl..

[7] Wäger P.A., Hischier R. (2015). Life cycle assessment of post-consumer plastics production from waste electrical and electronic equipment (WEEE) treatment residues in a Central European plastics recycling plant. Sci. Total Environ..

[8] Zhao Y.B., Lv X.D., Ni H.G. (2018). Solvent-based separation and recycling of waste plastics: A review. Chemosphere.

[9] Jacobsen R., Willeghems G., Gellynck X., Buysse J. (2018). Increasing the quantity of separated post-consumer plastics for reducing combustible household waste: The case of rigid plastics in Flanders. Waste Manag..

[10] Webb H.K., Arnott J., Crawford R.J., Ivanova E.P. (2013). Plastic degradation and its environmental implications with special reference to poly(ethylene terephthalate). Polymers.

[11] Ragaert K., Delva L., van Geem K. (2017). Mechanical and chemical recycling of solid plastic waste. Waste Manag..

[12] Faussone G.C. (2018). Transportation fuel from plastic: Two cases of study. Waste Manag..

[13] Kunwar B., Cheng H.N., Chandrashekaran S.R., Sharma B.K. (2016). Plastics to fuel: A review. Renew. Sustain. Energy Rev..

[14] Adibah W., Mahari W., TungChong C., KuiCheng C., LeingLee C., KristianHendrata, Yek P.N., LingMa N., ShiungLam S. (2018). Production of value-added liquid fuel via microwave co-pyrolysis of used frying oil and plastic waste. Energy.

[15] Miandad R., Barakat M.A., Aburiazaiza A.S., Rehan M., Ismail I.M.I., Nizami A.S. (2017). Effect of plastic waste types on pyrolysis liquid oil. Int. Biodeterior. Biodegrad..

[16] Verma R., Vinoda K.S., Papireddy M., Gowda A.N.S. (2016). Toxic pollutants from plastic waste- A Review. Procedia Environ. Sci..

[17] O’Brien J., Thondhlana G. (2019). Plastic bag use in South Africa: Perceptions, practices and potential intervention strategies. Waste Manag..

[18] Rivers N., Shenstone-Harris S., Young N. (2017). Using nudges to reduce waste? The case of Toronto’s plastic bag levy. J. Environ. Manag..

[19] Wagner T.P. (2017). Reducing single-use plastic shopping bags in the USA. Waste Manag..

[20] Martinho G., Balaia N., Pires A. (2017). The Portuguese plastic carrier bag tax: The effects on consumers’ behavior. Waste Manag..

[21] Asmuni S., Hussin N.B., Khalili J.M., Zain Z.M. (2015). Public Participation and Effectiveness of the no Plastic Bag Day Program in Malaysia. Procedia-Soc. Behav. Sci..

[22] Jakovcevic A., Steg L., Mazzeo N., Caballero R., Franco P., Putrino N., Favara J. (2014). Charges for plastic bags: Motivational and behavioral effects. J. Environ. Psychol..

[23] Vázquez-Morillas A., Velasco-Pérez M., Espinosa-Valdemar R.M., Morales-Contreras M., Hernández-Islas S., Ordaz-Guillén M.Y.L., Almeida-Filgueira H.J. (2016). Generación, legislación y valorización de residuos plásticos en Iberoamérica. Rev. Int. Contam. Ambient..

[24] Ohtomo S., Ohnuma S. (2014). Psychological interventional approach for reduce resource consumption: Reducing plastic bag usage at supermarkets. Resour. Conserv. Recycl..

[25] Poortinga W., Whitmarsh L., Suffolk C. (2013). The introduction of a single-use carrier bag charge in Wales: Attitude change and behavioural spillover effects. J. Environ. Psychol..

[26] Zhu Q. (2011). An appraisal and analysis of the law of ‘Plastic-Bag Ban’. Energy Procedia.

[27] Njeru J. (2006). The urban political ecology of plastic bag waste problem in Nairobi, Kenya. Geoforum.

[28] Siegenthaler K.O., Künkel A., Skupin G., Yamamoto M. (2012). Ecoflex® and Ecovio®: Biodegradable, Performance-Enabling Plastics. Advances in Polymer Science.

[29] ASTM International (2017). ASTM D883-17 Standard Terminology Relating to Plastics.

[30] Degli-Innocenti F., Chiellini E., Solaro R. (2002). Biodegradability and compostability. Biodegradable Polymers and Plastics.

[31] ASTM D6400-04 (2009). ASTM D 6400-04 Standard Specification for Compostable Plastics.

[32] EN 13432 (2000). Packaging-Requirements for Packaging Recoverable Through Composting and Biodegradation. Test Scheme and Evaluation Criteria for the Final Acceptance of Packaging.

[33] (2016). GESAMP Sources, fate and effects of microplastics in the marine environment: Part 2 of a global assessment. Rep. Stud. Jt. Group Expertes Sci. Asp. Mar. Environ. Protecion.

[34] Andrady A.L. (2011). Microplastics in the marine environment. Mar. Pollut. Bull..

[35] Vroom R.J.E., Koelmans A.A., Besseling E., Halsband C. (2017). Aging of microplastics promotes their ingestion by marine zooplankton. Environ. Pollut..

[36] Bellas J., Martínez-Armental J., Martínez-Cámara A., Besada V., Martínez-Gómez C. (2016). Ingestion of microplastics by demersal fish from the Spanish Atlantic and Mediterranean coasts. Mar. Pollut. Bull..

[37] Brennecke D., Duarte B., Paiva F., Caçador I., Canning-Clode J. (2016). Microplastics as vector for heavy metal contamination from the marine environment. Estuar. Coast. Shelf Sci..

[38] Kedzierski M., D’Almeida M., Magueresse A., le Grand A., Duval H., César G., Sire O., Bruzaud S., Le Tilly V. (2018). Threat of plastic ageing in marine environment. Adsorption/desorption of micropollutants. Mar. Pollut. Bull..

[39] Koelmans A.A., Besseling E., Foekema E.M. (2014). Leaching of plastic additives to marine organisms. Environ. Pollut.

[40] Hermabessiere L., Dehaut A., Paul-Pont I., Lacroix C., Jezequel R., Soudant P., Duflos G. (2017). Occurrence and effects of plastic additives on marine environments and organisms: A review. Chemosphere.

[41] Eerkes-Medrano D., Thompson R. (2018). Occurrence, Fate, and Effect of Microplastics in Freshwater Systems.

[42] van Wijnen J., Ragas A.M.J., Kroeze C. (2019). Modelling global river export of microplastics to the marine environment: Sources and future trends. Sci. Total Environ..

[43] de Souza Machado A.A., Lau C.W., Till J., Kloas W., Lehmann A., Becker R., Rillig M.C. (2018). Impacts of microplastics on the soil biophysical environment. Environ. Sci. Technol..

[44] Rillig M.C., Lehmann A., Machado A.A.S., Yang G. (2019). Microplastic effects on plants. New Phytol..

[45] Gasperi J., Wright S.L., Dris R., Collard F., Mandin C., Guerrouache M., Langlois V., Kelly F.J., Tassi B. (2018). Microplastics in air: Are we breathing it in?. Curr. Opin. Environ. Sci. Heal..

[46] Wright S.L., Kelly F.J. (2017). Plastic and Human Health: A Micro Issue?. Environ. Sci. Technol..

[47] Rujnić-Sokele M., Pilipović A. (2017). Challenges and opportunities of biodegradable plastics: A mini review. Waste Manag. Res..

[48] Rena P.G., Kumar S., Kumar S., Kumar R., Pandey A. (2019). Chapter 6-Landfill Gas as an Energy Source. Current Developments in Biotechnology and Bioengineering.

[49] Ji C., Kong C.X., Mei Z.L., Li J. (2017). A Review of the Anaerobic Digestion of Fruit and Vegetable Waste. Appl. Biochem. Biotechnol..

[50] Yoon Y.M., Kim S.H., Oh S.Y., Kim C.H. (2014). Potential of anaerobic digestion for material recovery and energy production in waste biomass from a poultry slaughterhouse. Waste Manag..

[51] Dadaser-Celik F., Azgin S.T., Yildiz Y.S. (2016). Optimization of solid content, carbon/nitrogen ratio and food/inoculum ratio for biogas production from food waste. Waste Manag. Res..

[52] Šan I., Onay T.T. (2001). Impact of various leachate recirculation regimes on municipal solid waste degradation. J. Hazard. Mater..

[53] Cuartas M., López A., Pérez F., Lobo A. (2018). Analysis of landfill design variables based on scientific computing. Waste Manag..

[54] Hernández-Berriel M.C., Márquez-Benavides L., González-Pérez D.J., Buenrostro-Delgado O. (2008). The effect of moisture regimes on the anaerobic degradation of municipal solid waste from Metepec (México). Waste Manag..

[55] Lv S., Zhang Y., Tan H. (2019). Thermal and thermo-oxidative degradation kinetics and characteristics of poly (lactic acid) and its composites. Waste Manag..

[56] Jamaluddin N., Razaina M.T., Ishak Z.A.M. (2016). Mechanical and Morphology Behaviours of Polybutylene (succinate)/Thermoplastic Polyurethaneblend. Procedia Chem..

[57] Shi X.Q., Ito H., Kikutani T. (2005). Characterization on mixed-crystal structure and properties of poly(butylene adipate-co-terephthalate) biodegradable fibers. Polym. Guildf.

[58] Khanal S.K. (2008). Anaerobic Biotechnology for Bioenergy Production: Principles and Applications.

[59] Siles J.A., Brekelmans J., Martín M.A., Chica A.F., Martín A. (2010). Impact of ammonia and sulphate concentration on thermophilic anaerobic digestion. Bioresour. Technol..

[60] Abdul-Sattar N., Caruana D.J., Olsen A.E. (2012). Anaerobic digestion: Processes, products and applications. Anaerobic Disgestion.

[61] Mohee R., Unmar G.D., Mudhoo A., Khadoo P. (2008). Biodegradability of biodegradable/degradable plastic materials under aerobic and anaerobic conditions. Waste Manag..

[62] Gutierrez-Wing M.T., Stevens B.E., Theegala C.S., Negulescu I.I., Rusch K.A. (2010). Anaerobic biodegradation of polyhydroxybutyrate in municipal sewage sludge. J. Environ. Eng..

[63] Mezzanotte V., Bertani R., Innocenti F.D., Tosin M. (2005). Influence of inocula on the results of biodegradation tests. Polym. Degrad. Stab..

[64] Vaverková D.A.M. (2014). Degradation of biodegradable/degradable plastics in municipal solid-waste landfill. Polish J. Environ. Stud..

[65] Ishigaki T., Sugano W., Nakanishi A., Tateda M., Ike M., Fujita M. (2004). The degradability of biodegradable plastics in aerobic and anaerobic waste landfill model reactors. Chemosphere.

[66] Landreth R. (2002). Use of Municipal Solid Waste Landfills as Biochemical Reactors. U.S..

[67] Cossu R., Morello L., Stegmann R., Cossu R., Stegmann R. (2018). 3.1-Biochemical Processes in Landfill. Solid Waste Landfilling.

[68] Batarseh E.S., Reinhart D.R., Berge N.D. (2010). Sustainable disposal of municipal solid waste: Post bioreactor landfill polishing. Waste Manag..

[69] Raichev R., Veleva L., Valdez B. (2009). Corrosión de metales y degradación de materiales. Principios y Prácticas de Laboratorio.

[70] Singh B., Sharma N. (2008). Mechanistic implications of plastic degradation. Polym. Degrad. Stab..

[71] Aguilar L. (2011). Evaluación del Deterioro de Polímero Sellante de Juntas de Motor en un Ambiente con Mezclas de Etanol-Gasolina. Bachelor’s Thesis.

[72] Carballa M., Manterola G., Larrea L., Ternes T., Omil F., Lema J.M. (2007). Influence of ozone pre-treatment on sludge anaerobic digestion: Removal of pharmaceutical and personal care products. Chemosphere.

[73] Adamcová D., Vaverková M.D. (2016). New polymer behavior under the landfill conditions. Waste Biomass Valorization.

[74] Goómez E.F., Luo X., Li C., Michel F.C., Yebo L. (2014). Biodegradability of crude glycerol-based polyurethane foams during composting, anaerobic digestion and soil incubation. Polym. Degrad. Stabilit.

[75] Lima V., Hossain U.H., Walbert T., Seidl T., Ensinger W. (2018). Mass spectrometric comparison of swift heavy ion-induced and anaerobic thermal degradation of polymers. Radiat. Phys. Chem..

[76] Vernáez O., Dagreou S., Grassl B., Müller A.J. (2015). Degradation of styrene butadiene rubber (SBR) in anaerobic conditions. Polym. Degrad. Stab..

[77] Gorrasi G., Pantani R. (2018). Hydrolysis and Biodegradation of Poly(lactic acid). Advances in Polymer Science.

[78] Ryan C.A., Billington S.L., Criddle C.S. (2017). Methodology to assess end-of-life anaerobic biodegradation kinetics and methane production potential for composite materials. Compos. Part. A Appl. Sci. Manuf..

[79] Arvanitoyannis I., Biliaderis C.G., Ogawa H., Kawasaki N. (1998). Biodegradable films made from low-density polyethylene (LDPE), rice starch and potato starch for food packaging applications: Part 1. Carbohydr. Polym..

[80] Xing W., Lu W., Zhao Y., Zhang X., Deng W., Christensen T.H. (2013). Environmental impact assessment of leachate recirculation in landfill of municipal solid waste by comparing with evaporation and discharge (EASEWASTE). Waste Manag..

[81] Feng S.J., Chen Z.W., Chen H.X., Zheng Q.T., Liu R. (2018). Slope stability of landfills considering leachate recirculation using vertical wells. Eng. Geol..

[82] Jain P., HacKo J., Kumar D., Powell J., Kim H., Maldonado L., Townsend T., Reinhart D.R. (2014). Case study of landfill leachate recirculation using small-diameter vertical wells. Waste Manag..

[83] Dvorackova M., Svoboda P., Kostka L., Pekarova S. (2015). Influence of biodegradation in thermophilic anaerobic aqueous conditions on crystallization of poly(butylene succinate). Polym. Test..

[84] Iwańczuk A., Kozłowski M., Łukaszewicz M., Jabłoński S. (2015). Anaerobic biodegradation of polymer composites filled with natural fibers. J. Polym. Environ..

[85] Yagi H., Ninomiya F., Funabashi M., Kunioka M. (2013). Thermophilic anaerobic biodegradation test and analysis of eubacteria involved in anaerobic biodegradation of four specified biodegradable polyesters. Polym. Degrad. Stab..

[86] Yagi H., Ninomiya F., Funabashi M., Kunioka M. (2014). Mesophilic anaerobic biodegradation test and analysis of eubacteria and archaea involved in anaerobic biodegradation of four specified biodegradable polyesters. Polym. Degrad. Stab..

[87] Yagi H., Ninomiya F., Funabashi M., Kunioka M. (2009). Anaerobic biodegradation tests of poly(lactic acid) and polycaprolactone using new evaluation system for methane fermentation in anaerobic sludge. Polym. Degrad. Stab..

[88] Boonmee J., Kositanont C., Leejarkpai T. (2016). Biodegradation of poly (lactic acid), poly (hydroxybutyrate-co-hydroxyvalerate), poly (butylene succinate) and poly(butylene adipate-co-terephthalate) under anaerobic and oxygen limited thermophilic conditions. Environ. Asia.

[89] Abou-Zeid D.-M., Müller R.-J., Deckwer W.D. (2001). Degradation of natural and syntehtic polyesters under anaerobic conditions. J. Biotechnol..

[90] Nauendorf A., Krause S., Bigalke N.K., Gorb E.V., Gorb S.N., Haeckel M., Wahl M., Treude T. (2016). Microbial colonization and degradation of polyethylene and biodegradable plastic bags in temperate fine-grained organic-rich marine sediments. Mar. Pollut. Bull..

[91] Rivard C., Moens L., Roberts K., Brigham J., Kelley S. (1995). Starch esters as biodegradable plastics: Effects of ester group chain length and degree of substitution on anaerobic biodegradation. Enzyme Microb. Technol..

[92] Halley P.J., Truss R.W., Markotsis M.G., Chaleat C., Russo M., Sargent A.L., Tan I., Sopade P.A. (2007). A Review of Biodegradable Thermoplastic Starch Polymers. Polymer Durability and Radiation Effects.

[93] Mackuľak T., Takáčová A., Gál M., Marton M., Ryba J. (2015). PVC degradation by fenton reaction and biological decomposition. Polym. Degrad. Stab..

[94] Morse M.C., Liao Q., Criddle C.S., Frank C.W. (2011). Anaerobic biodegradation of the microbial copolymer poly(3-hydroxybutyrate-co-3-hydroxyhexanoate): Effects of comonomer content, processing history, and semi-crystalline morphology. Polym. Guildf.

[95] McDevitt J.P., Criddle C.S., Morse M., Hale R.C., Bott C.B., Rochman C.M. (2017). Addressing the Issue of Microplastics in the Wake of the Microbead-Free Waters Act - A New Standard Can Facilitate Improved Policy. Environ. Sci. Technol..

[96] ASTM International (2018). ASTM D5511-18 Standard Test Method for Determining Anaerobic Biodegradation of Plastic Materials Under High-Solids Anaerobic-Digestion Conditions.

[97] ASTM International (2011). ASTM D7475-11 Standard Test Method for Determining the Aerobic Degradation and Anaerobic Biodegradation of Plastic Materials Under Accelerated Bioreactor Landfill Conditions.

[98] ASTM International (2012). ASTM D5526-12 Standard Test Method for Determining the Aerobic Degradation and Anaerobic Biodegradation of Plastic Materials Under Accelerated Bioreactor Landfill Conditions.

[99] ISO 15985:2014(en), Plastics—Determination of the Ultimate Anaerobic Biodegradation under High-Solids Anaerobic-Digestion Conditions—Method by Analysis of Released Biogas. [En línea]. https://www.iso.org/obp/ui/#iso:std:iso:15985:ed-2:v1:en.

[100] ISO 13975:2019(en), Plastics—Determination of the Ultimate Anaerobic Biodegradation of Plastic Materials in Controlled Slurry Digestion Systems—Method by Measurement of Biogas Production. [En línea]. https://www.iso.org/obp/ui/#iso:std:iso:13975:ed-2:v1:en.

[101] ISO 14853:2016(en), Plastics—Determination of the Ultimate Anaerobic Biodegradation of Plastic Materials in an Aqueous System—Method by Measurement of Biogas Production. [En línea]. https://www.iso.org/obp/ui/#iso:std:iso:14853:ed-2:v1:en.

[102] Day M., Shaw K., Cooney D. (1994). Biodegradability: An assessment of commercial polymers according to the Canadian method for anaerobic conditions. J. Environ. Polym. Degrad..

[103] Yagi H., Ninomiya F., Funabashi M., Kunioka M. (2012). Anaerobic Biodegradation of Poly (Lactic Acid) Film in Anaerobic Sludge. J. Polym. Environ..

[104] Massardier-Nageotte V., Pestre C., Cruard-Pradet T., Bayard R. (2006). Aerobic and anaerobic biodegradability of polymer films and physico-chemical characterization. Polym. Degrad. Stab..

[105] Lee J.C., Moon J.H., Jeong J.-H., Kim M.Y., Kim B.M., Choi M.-C., Kim J.R. (2016). Biodegradability of poly(lactic acid) (PLA)/lactic acid (LA) blends using anaerobic digester sludge. Macromol. Res..

[106] Selke S., Auras R., Nguyen T.A., Castro Aguirre E., Cheruvathur R., Liu Y. (2015). Evaluation of biodegradation-promoting additives for plastics. Environ. Sci. Technol..

[107] Hermanová S., Šmejkalová P., Merna J., Zarevúcka M. (2015). Biodegradation of waste PET based copolyesters in thermophilic anaerobic sludge. Polym. Degrad. Stab..

[108] Wang F., Hidaka T., Tsuno H., Tsubota J. (2012). Co-digestion of polylactide and kitchen garbage in hyperthermophilic and thermophilic continuous anaerobic process. Bioresour. Technol..

[109] Soda S., Iwama K., Yokoe K., Okada Y., Ike M. (2016). High methane production potential of activated sludge accumulating polyhydroxyalkanoates in anaerobic digestion. Biochem. Eng. J..

[110] Petit M.G., Correa Z., Sabino M.A. (2015). Degradation of a Polycaprolactone/Eggshell Biocomposite in a Bioreactor. J. Polym. Environ..

[111] Xiao D., Matsuda J., Liu B., Ohmiya K. (2009). Characteristics of fermentation of biodegradable plastics mixed with household solid waste by thermophilic dry anaerobic co-digestion. J. Jpn. Soc. Agric. Mach..

[112] Shin P.K., Kim M.H., Kim J.M. (1997). Biodegradability of degradable plastics exposed to anaerobic digested sludge and simulated landfill conditions. J. Environ. Polym. Degrad..

[113] Yagi H., Ninomiya F., Funabashi M., Kunioka M. (2009). Anaerobic biodegradation tests of poly(lactic acid) under mesophilic and thermophilic conditions using a new evaluation system for methane fermentation in anaerobic sludge. Int. J. Mol. Sci..

[114] Shi B., Palfery D. (2010). Enhanced mineralization of PLA meltblown materials due to plasticization. J. Polym. Environ..

[115] Rivard C.J., Adney W.S., Himmel M.E., Mitchell D.J., Vinzant T.B., Grohmann K., Moens L., Chum H. (1992). Effects of natural polymer acetylation on the anaerobic bioconversion to methane and carbon dioxide. Appl. Biochem. Biotechnol..

[116] Lim J.W., Ting D.W.Q., Loh K.C., Ge T., Tong Y.W. (2018). Effects of disposable plastics and wooden chopsticks on the anaerobic digestion of food waste. Waste Manag..

[117] Vázquez-Morillas A., Hermoso-López Araiza J.P., Álvarez-Zeferino J.C., Beltrán-Villavicencio M., Espinosa-Valdemar R.M., Quecholac-Piña X., Sotelo-Navarro P.X., Velasco-Pérez M. (2017). Utilities such as purchase bags, goods packaging and composting bags. Green Polymer Composites Technology.

[118] Shanks R., Kong I., El-Sonbati P.A. (2012). Thermoplastic Starch. Thermoplastic Elastomers.

[119] Krupp L.R., Jewell W.J. (1992). Biodegradability of modified plastic films in controlled biological environments. Environ. Sci. Technol..

[120] Breslin V.T. (1993). Degradation of starch-plastic composites in a municipal solid waste landfill. J. Environ. Polym. Degrad..

[121] Zhang W., Heaven S., Banks C.J. (2018). Degradation of some EN13432 compliant plastics in simulated mesophilic anaerobic digestion of food waste. Polym. Degrad. Stab..

[122] Weiwei L., Juan X., Beijiu C., Suwen Z., Qing M., Huan M. (2016). Anaerobic biodegradation, physical and structural properties of normal and high-amylose maize starch films. Int. J. Agric. Biol. Eng..

[123] Gómez E.F., Michel F.C. (2013). Biodegradability of conventional and bio-based plastics and natural fiber composites during composting, anaerobic digestion and long-term soil incubation. Polym. Degrad. Stab..

[124] Cho H.S., Moon H.S., Kim M., Nam K., Kim J.Y. (2011). Biodegradability and biodegradation rate of poly(caprolactone)-starch blend and poly(butylene succinate) biodegradable polymer under aerobic and anaerobic environment. Waste Manag..

[125] Šmejkalová P., Kužníková V., Merna J., Hermanová S. (2016). Anaerobic digestion of aliphatic polyesters. Water Sci. Technol..

[126] Budwill K., Fedorak P.M., Page W.J. (1992). Methanogenic degradation of poly(3-hydroxyalkanoates). Appl. Environ. Microbiol..

[127] Ryan C.A., Billington S.L., Criddle C.S. (2017). Assessment of models for anaerobic biodegradation of a model bioplastic: Poly(hydroxybutyrate-co-hydroxyvalerate). Bioresour. Technol..

[128] Reischwitz A., Stoppok E., Buchholz K. (1997). Anaerobic degradation of poly-3-hydroxybutyrate and poly-3-hydroxybutyrate-co-3-hydroxyvalerate. Biodegradation.

[129] Kolstad J.J., Vink E.T.H., de Wilde B., Debeer L. (2012). Assessment of anaerobic degradation of Ingeo™ polylactides under accelerated landfill conditions. Polym. Degrad. Stab..

[130] Yagi H., Ninomiya F., Funabashi M., Kunioka M. (2011). RNA analysis of anaerobic sludge during anaerobic biodegradation of cellulose and poly(lactic acid) by RT-PCR–DGGE. Polym. Degrad. Stab..

[131] Wang F., Tsuno H., Hidaka T., Tsubota J. (2011). Promotion of polylactide degradation by ammonia under hyperthermophilic anaerobic conditions. Bioresour. Technol..

[132] Yagi H., Ninomiya F., Funabashi M., Kunioka M. (2010). Bioplastic biodegradation activity of anaerobic sludge prepared by preincubation at 55°C for new anaerobic biodegradation test. Polym. Degrad. Stab..

[133] Vargas L.F., Welt B.A., Teixeira A., Pullammanappallil P., Balaban M., Beatty C. (2009). Biodegradation of treated polylactic acid (PLA) under anaerobic conditions. Am. Soc. Agric. Biol. Eng..

[134] Guo M., Trzcinski A.P.P., Stuckey D.C.C., Murphy R.J.J. (2011). Anaerobic digestion of starch–polyvinyl alcohol biopolymer packaging: Biodegradability and environmental impact assessment. Bioresour. Technol..

[135] Urgun-Demirtas M., Singh D., Pagilla K. (2007). Laboratory investigation of biodegradability of a polyurethane foam under anaerobic conditions. Polym. Degrad. Stab..

[136] Tollner E.W., Annis P.A., Das K.C. (2011). Evaluation of Strength Properties of Polypropylene-Based Polymers in Simulated Landfill and Oven Conditions. J. Environ. Eng..

[137] Björn A., Hörsing M., Karlsson A., Mersiowsky I., Ejlertsson J. (2007). Impacts of temperature on the leaching of organotin compounds from poly(vinyl chloride) plastics-A study conducted under simulated landfill conditions. J. Vinyl Addit. Technol..

