# Supplementary material for: Degradation of Plastics under Anaerobic Conditions: A Short Review
Source: Polymers (Basel). 2020 Jan 5;12(1):109. doi: 10.3390/polym12010109 (PMC7023122; doi:10.3390/polym12010109)
Supplement: Supplementary file 1 [file polymers-12-00109-s001.pdf]

Supporting Information

Degradation of Plastics under Anaerobic Conditions: A Short Review

Xochitl Quecholac-Piña [1], María del Consuelo Hernández-Berriel [1], María del Consuelo Mañón-Salas [2], Rosa María Espinosa-Valdemar [3] and Alethia Vázquez-Morillas [3,*]

[1] Tecnológico Nacional de México/Instituto Tecnológico de Toluca, Av. Tecnológico s/n. Colonia Agrícola Bellavista Metepec, Edo. De México, México C.P. 52149, México; xquecholacp@toluca.tecnm.mx (X.Q.-P.); mhernandezb@toluca.tecnm.mx (M.d.C.H.-B.)

[2] Sociedad Mexicana de Ciencia y Tecnología Aplicada a Residuos Sólidos, A.C., Priv Molcajete 44 Fracc. Hacienda de las fuentes, Calimaya, México C.P. 52227, México; consuelomanon@gmail.com

[3] Universidad Autónoma Metropolitana, Av San Pablo Xalpa 180, Reynosa Tamaulipas, Azcapotzalco, Ciudad de México 02200, México; rmev@azc.uam.mx

* Correspondence: alethia@azc.uam.mx; Tel.: +52-55-53189057

Table S1. List of articles.

No.	List of Articles	Reference
1	Degradation of some EN13432 compliant plastics in simulated mesophilic anaerobic digestion of food waste	[1]
2	Biodegradation of poly(3-hydroxybutyrate-co-3-hydroxyhexanoate) plastic under anaerobic sludge and aerobic seawater conditions: gas evolution and microbial diversity	[2]
3	Effects of disposable plastics and wooden chopsticks on the anaerobic digestion of food waste	[3]
4	Biocomposite fiber-matrix treatments that enhance in-service performance can also accelerate end-of-life fragmentation and anaerobic biodegradation to methane	[4]
5	The characteristic changes of rice straw fibers in anaerobic digestion and its effect on rice straw-reinforced composite	[5]
6	Methodology to assess end-of-life anaerobic biodegradation kinetics and methane production potential for composite materials	[6]
7	Assessment of models for anaerobic biodegradation of a model bioplastic: Poly(hydroxybutyrate-co-hydroxyvalerate)	[7]
8	Estimation of the microbial degradation of biodegradable polymer, poly(lactic acid) (PLA) with a specific gas production rate	[8]
9	Biodegradation of poly(Lactic acid), poly(hydroxybutyrate-co-hydroxyvalerate), poly(butylene succinate) and poly(butylene adipate-co-terephthalate) under anaerobic and oxygen limited thermophilic conditions	[9]
10	New polymer behavior under the landfill conditions	[10]
11	Anaerobic digestion of aliphatic polyesters	[11]
12	Anaerobic biodegradation, physical and structural properties of normal and high-amylose maize starch films	[12]
13	Biodegradability of poly(lactic acid) (PLA)/lactic acid (LA) blends using anaerobic digester sludge	[13]
14	Microbial colonization and degradation of polyethylene and biodegradable plastic bags in temperate fine-grained organic-rich marine sediments	[14]
15	PVC degradation by Fenton reaction and biological decomposition	[15]
16	Influence of biodegradation in thermophilic anaerobic aqueous conditions on crystallization of poly(butylene succinate)	[16]
17	Degradation of a Polycaprolactone/Eggshell Biocomposite in a Bioreactor	[17]
18	Microbial consortium involving biological methane oxidation in relation to the biodegradation of waste plastics in a solid waste disposal open dump site	[18]
19	Anaerobic biodegradation of polymer composites filled with natural fibers	[19]
20	Evaluation of biodegradation-promoting additives for plastics	[20]
21	Biodegradation of waste PET based co polyesters in thermophilic anaerobic sludge	[21]
22	Degradation of biodegradable/degradable plastics in municipal solid-waste landfill	[22]

No.	List of Articles	Reference
23	Biodegradability of crude glycerol-based polyurethane foams during composting, anaerobic digestion and soil incubation	[23]
24	Comparative oxo-biodegradation study of poly-3-hydroxybutyrate-co- 3-hydroxyvalerate/polypropylene blend in controlled environments	[24]
25	Mesophilic anaerobic biodegradation test and analysis of eubacteria and archaea involved in anaerobic biodegradation of four specified biodegradable polyesters	[25]
26	Biodegradability of conventional and bio-based plastics and natural fiber composites during composting, anaerobic digestion and long-term soil incubation	[26]
27	Thermophilic anaerobic biodegradation test and analysis of eubacteria involved in anaerobic biodegradation of four specified biodegradable polyesters	[27]
28	Assessment of anaerobic degradation of Ingeo™ polylactides under accelerated landfill conditions	[28]
29	Anaerobic biodegradation of poly (Lactic Acid) film in anaerobic sludge	[29]
30	Evaluation of strength properties of polypropylene-based polymers in simulated landfill and oven conditions	[30]
31	Biodegradability and biodegradation rate of poly (caprolactone)-starch blend and poly(butylene succinate) biodegradable polymer under aerobic and anaerobic environment	[31]
32	RNA analysis of anaerobic sludge during anaerobic biodegradation of cellulose and poly(lactic acid) by RT-PCR–DGGE	[32]
33	Anaerobic biodegradation of the microbial copolymer poly(3-hydroxybutyrate-co-3-hydroxyhexanoate): effects of comonomer content, processing history, and semi-crystalline morphology	[33]
34	Anaerobic digestion of starch–polyvinyl alcohol biopolymer packaging: Biodegradability and environmental impact assessment	[34]
35	Promotion of poly lactide degradation by ammonia under hyperthermophilic anaerobic conditions	[35]
36	Enhanced mineralization of PLA meltblown materials due to plasticization	[36]
37	Bioplastic biodegradation activity of anaerobic sludge prepared by pre-incubation at 55°C for new anaerobic biodegradation test	[37]
38	Biodegration of treated polylactic acid (PLA) under anaerobic conditions	[38]
39	Anaerobic biodegradation of polyhydroxybutyrate in municipal sewage sludge	[39]
40	The anaerobic degradability of thermoplastic starch: Polyvinyl alcohol blends: Potential biodegradable food packaging materials	[40]
41	Characteristics of fermentation of biodegradable plastics mixed with household solid waste by thermophilic dry anaerobic co-digestion	[41]
42	Anaerobic biodegradation tests of poly(lactic acid) under mesophilic and thermophilic conditions using a new evaluation system for methane fermentation in anaerobic sludge	[42]
43	Anaerobic biodegradation tests of poly(lactic acid) and polycaprolactone using new evaluation system for methane fermentation in anaerobic sludge	[43]
44	Determining biodegradability of polylactic acid under diferent enviroments	[44]
45	Biodegradability of biodegradable/degradable plastic materials under aerobic and anaerobic conditions	[45]
46	Laboratory investigation of biodegradability of a polyurethane foam under anaerobic conditions	[46]
47	Considerations Affecting Landfill Biodegradability of PVC	[47]
48	Impacts of temperature on the leaching of organotin compounds from poly(vinyl chloride) plastics-A study conducted under simulated landfill conditions	[48]
49	Aerobic and anaerobic biodegradability of polymer films and physico-chemical characterization	[49]
50	Biodegradation of aliphatic homopolyesters and aliphatic-aromatic copolyesters by anaerobic microorganisms	[50]
51	The degradability of biodegradable plastics in aerobic and anaerobic waste landfill model reactors	[51]
52	Anaerobic biodegradation of aliphatic polyesters: poly(3-hydroxybutyrate-co-3-hydroxyoctanoate) and poly(e-caprolactone)	[52]
53	Degradation of natural and synthetic polyesters under anaerobic conditions	[53]
54	Fate of plasticised PVC products under Landfill conditions: a laboratory-scale landfill simulation reactor study	[54]
55	Long term behaviour of poly(vinyl chloride) products under soil buried and landfill conditions	[55]
56	Migration and release profile of chimassorb 944 from low-density	[56]

No.	List of Articles	Reference
57	Biodegradable films made from low-density polyethylene (LDPE), rice starch and potato starch for food packaging applications: Part 1	[57]
58	Testing anaerobic biodegradability of polymers in a laboratory-scale simulated landfill	[58]
59	Biodegradable films made from low density polyethylene (LDPE), wheat starch and soluble starch for food packaging applications. Part 2	[59]
60	Anaerobic degradation of poly-3-hydroxybutyrate and poly-3-hydroxybutyrate-co-3-hydroxyvalerate	[60]
61	Biodegradability of degradable plastics exposed to anaerobic digested sludge and simulated landfill conditions	[61]
62	Starch esters as biodegradable plastics: effects of ester group chain length and degree of substitution on anaerobic biodegradation	[62]
63	Biodegradation of poly-β-hydroxyalkanoates in anaerobic sediments	[63]
64	Biodegradability: an assessment of commercial polymers according to the Canadian method for anaerobic conditions	[64]
65	Confirmation of anaerobic poly(2-oxepanone) degrading microorganisms in environments	[65]
66	Assessment of biodegradation of water insoluble polymeric materials in aerobic and anaerobic aquatic environments	[66]
67	Degradation of starch-plastic composites in a municipal solid waste landfill	[67]
68	Methanogenic degradation of poly(3-hydroxyalkanoates)	[68]
69	Effects of natural polymer acetylation on the anaerobic bioconversion to methane and carbon dioxide	[69]
70	Biodegradability of modified plastic films in controlled biological environments	[70]

Table S2. Summary of experimental conditions and results.

Reference	Type plastic	Degradation	Co-sustrate	Working volume	Temperature	Duration	Scale	% Biodegradation	Biogas
[1]	Polypropylene<br>Low density polyethylene<br>Cellulose-based metallized<br>Cellulose-based heat- sealable<br>Cellulose-based non heat-sealable<br>Starch-based film blend 1<br>Starch-based film blend 2<br>Poly lactic Acid Film	Anaerobic digestion	Post-consumer domestic food waste and card packaging at a ratio of 80:20% on a fresh weight basis	4.5 L	37 °C	147 day	Laboratory	-	-
[2]	Poly (3-hydroxybutyrate-co-3-hydroxyhexanoate)	Anaerobic digestion	-	500 mL	38 °C	85 days	Laboratory	-	-
[3]	Polystyrene (PS)<br>Polypropylene (PP)<br>High density polyethylene (HDPE)<br>Wooden chopsticks (WC)	Anaerobic digestion	Food waste	1000 mL	35 °C	30-35 days	Laboratory	-	-
[4]	Poly (hydroxybutyrate-co-hydroxyvalerate) (PHBV)<br><br>Poly (hydroxybutyrate-co-hydroxyvalerate) (PHBV) with wood fiber (WF)	Anaerobic digestion	-	600 mL	37 °C	84 days	Laboratory	-	-
[5]	Rice straw/low-density polyethylene (RS/LDPE)	Anaerobic digestion		5 L	37 °C	30 days	Laboratory	-	71800 mL
[6]	Poly (hydroxybutyrate- co-hydroxyvalerate) (PHBV) Composites with wood fiber (WF) (0%, 20%, 40%)	Anaerobic digestion		600 mL	37 °C	56 days	Laboratory	-	-
[7]	Poly (hydroxybutyrate-co-hydroxyvalerate) (PHBV)	Anaerobic digestion	-	600 mL	37 °C	42 days	Laboratory	86%	-
[8]	Poly (lactic acid) (PLA)	Anaerobic digestion	40 mM acetate was augmented in phase I, 4 mM in phase II and finally 0 mM in phase III	250 mL	37 °C	30 days	Laboratory	-	0.35 mmol/day m^2

Reference	Type plastic	Degradation	Co-sustrate	Working volume	Temperature	Duration	Scale	% Biodegradation	Biogas
[9]	Poly (lactic acid) (PLA), Poly (hydroxybutyrate-co-hydroxyvalerate) (PHBV), Poly (butylene succinate) (PBS), and poly (butylene adipate-co-terephthalate) (PBAT)	Landfill	Waste solid	660 mL	52 °C	75 days	Laboratory	-	-
[10]	High-density polyethylene (HDPE) with the totally degradable plastic additives (TDPA)<br>Polyethylene (PE) labeled as 100% degradable<br>Compostable Starch and Polycaprolactone	Landfill	Municipal waste	Real	6.2 -20.8	2 years	Landfill	-	-
[11]	Poly (L-lactic acid) (PLA)<br>Poly (ε-caprolactone) (PCL)	Anaerobic digestion	-	120 mL	55 °C	150 days	Laboratory	PCL 74%, PLA 62%	PLA 676–677 mL/g TS
[12]	Starch/polyvinyl alcohol (PVA) blend	Anaerobic digestion	-	1 L	35 °C	26 days	Laboratory	52.09% ± 0.95%	(7980 ± 20 mL)
[13]	Poly (lactic acid) (PLA)<br>PLA blend	Anaerobic digestion	-	250 mL	37 °C	30 days	Laboratory	-	0.0068 mmol/day m^2
[14]	Polyethylene (PE)<br>Biodegradable polyester	Anaerobic digestion	-	200 mL	-	98 days	Laboratory	-	-
[15]	Poly vinyl chloride (PVC)	Anaerobic digestion	-	650 mL	37–38 °C	7 days	Laboratory	-	7.7 mL
[16]	Poly (butylene succinate) (PBS)	Anaerobic digestion		250 mL /100 mL	55–37 °C	113 days	Laboratory	-	-
[17]	Polycaprolactone (PCL)/eggshell (ES) biocomposite (50/50 w/w)	Anaerobic digestion	-	-	43 °C	8 weeks	Laboratory	-	-
[18]	High/low density polyethylene, HDPE/LDPE<br>Poly- propylene, PP<br>Polystyrene, PS	Landfill	Stabilized organic wastes	Diameter of 5 cm and length of 150 cm	28–30 °C	357 days	Laboratory	-	-
[19]	Poly (lactic acid), polyhydroxybutyrate-co-polyhydroxyvalerate and low density polyethylene	Anaerobic digestion	-	120 mL	36 °C	28 days	Laboratory	-	936.6 ml/g PHB/PHV
[20]	Polyethylene (PE)	Anaerobic digestion	Household waste	125 mL	35 and 50 °C	464 days	Laboratory	-	-

Reference	Type plastic	Degradation	Co-sustrate	Working volume	Temperature	Duration	Scale	% Biodegradation	Biogas
	Polyethylene terephthalate (PET)								
[21]	Poly (ethylene terephthalate-co-lactate) copolyesters	Anaerobic digestion	-	120 mL	55 °C	394 days	Laboratory	-	-
[22]	Polyethylene sample with the additive<br>Polyethylene (PE) labeled as 100% degradable<br>Compostable<br>Starch and Polycaprolactone	Landfill	Waste solid	Real	3.4-20.58 °C	1 year	Real	No modification of structure	-
[23]	Polyurethane (PU) foams made from crude glycerol<br>Polyurethane (PU)<br>Petroleum- based polyols	Anaerobic digestion	-	2 L	37 °C	105 days	Laboratory	PU foams made from bio-based 8.95%<br>blend polyols 8.5%	-
[24]	Poly-3-hydroxybutyrate-co-3-hydroxyvalerate (PHBV) copolymer as a biodegradable additive in polypropylene (PP)	Anaerobic digestion	-	-	25, 35, 45, 55 °C	28 days	Laboratory	-	-
[25]	Poly (caprolactone) (PCL)<br>Poly (lactic acid) (PLA)<br>Polyhydroxybutyrate (PHB)<br>Poly (butylene succinate) (PBS)	Anaerobic digestion	-	1.5 L	37 °C	277 days	Laboratory	PCL: 12.5%<br>PLA: 39%<br>PHB: 92.5%<br>PBS: 0	2.07 L<br>4.05 L<br>10.61 L
[26]	Blend of polypropylene (PP) with 2% additive<br>Blend of polyethylene terephthalate (PETE) with 1% additive<br>Plastarch<br>Co-polyester þ corn-based plastic	Anaerobic digestion	the organic fraction of municipal solid waste	2 L	37 °C	50 days	Laboratory	Blend of polypropylene (PP) with 2% additive 3.1<br>Blend of polyethylene terephthalate (PETE) with 1% additive 2.2<br>Plastarch 26.4<br>Co-polyester þ corn-based plastic 20.2	-
[27]	Polycaprolactone (PCL)<br>Poly (lactic acid) (PLA)<br>Polyhydroxybutyrate (PHB)<br>Poly (butylene succinate) (PBS)	Anaerobic digestion	-	2 L	55 °C	50 days	Laboratory	PHB 90%<br>PCL 80%<br>PLA 75%	PHB 10.25 L<br>PCL 9.93 L<br>PLA 7.41 L<br>PBS 0.54 L

Reference	Type plastic	Degradation	Co-sustrate	Working volume	Temperature	Duration	Scale	% Biodegradation	Biogas
								PBS could not be anaerobically biodegraded	
[28]	Poly lactide (PLA) semicrystalline, one amorphous	Anaerobic digestion<br>Landfill	high amount of pre-treated municipal solid waste fraction	2 L	35 °C<br>21 °C	170 days<br>13 months	Laboratory	Anaerobic digestión : amorphous PLA 36%<br>Landfill: not biodegraded	-
[29]	Poly (lactic acid) (PLA	Anaerobic digestion	-	1.5 L	55 °C	57 days	Laboratory	77.25%	7.07 L
[30]	Polypropylene (PP)	Landfill	50% biosolids and 50% (volume basis) municipal solid waste (MSW)	208 L	35 °C, 45 °C, and 60 °C	52 weeks	Pilot	-	-
[31]	Poly (caprolac- tone) (PCL)-starch blend<br>Poly (butylene succinate) (PBS)	Anaerobic digestion<br>Landfill	-<br>Municipal solid waste	250 mL<br>Landfill	35 °C	139 days<br>90 days	Real landfill	PCL-starch blend 83% PBS 2%<br>Physical deterioration	669 mL-CH_4/g-VS<br>11 mL-CH_4/g-VS
[32]	poly(lactic acid) (PLA)	Anaerobic digestion	-	1.5 L	55 °C	45 days	Laboratory	70%	7.16 L
[33]	poly(3-hydroxybutyrate-co-3-hydroxyhexanoate)	Anaerobic digestion	-	50 mL	37 °C	12 days	Laboratory	95%	-
[34]	starch–polyvinyl alcohol (PVOH)	Anaerobic digestion	-	165 mL	37 °C	115 days	Laboratory	60%	-
[35]	Poly lactide (PLA) 100%<br>Poly lactide (PLA) 70%	Anaerobic digestion	Kitchen garbage and ammonia	1 L	80 °C and 55 °C	22 days	Laboratory	PLA (100%) 81.8%<br>PLA (70%) 77.0%	-
[36]	Poly lactic acid (PLA) with Poly (propylene glycol) (PPG)	Anaerobic digestion	-	-	35 and 50 °C	~182 days	Laboratory	90%	-
[37]	Poly (lactic acid) (PLA)	Anaerobic digestion	-	1.5 L	37 and 55 °C	100 days	Laboratory	91.5%	8.92
[38]	Poly lactic acid (PLA)<br>Gamma-irradiated	Anaerobic digestion	-	280 mL	37 and 58 °C	56 days	Laboratory	37 °C: 0.19%<br>58 °C: 98.96<br>*gamma-irradiated PLA lost 45%*	-
[39]	Poly hydroxybutyrate (PHB)	Anaerobic digestion	-	240 mL	35 °C	32 weeks	Laboratory	100	-
[40]	Starch: poly vinyl alcohol (TPS: PVOH)<br>w/w%: 90:10, 75:25, 50:50, 0:100	Anaerobic digestion	-	200 mL	38 °C	100 h	Laboratory	-	-

Reference	Type plastic	Degradation	Co-sustrate	Working volume	Temperature	Duration	Scale	% Biodegradation	Biogas
[41]	Poly lactide (PLA)-based Rubbish collection bags (50% PLA)<br>Fresh-keeping bags (90% PLA)<br>Drinking cups (80%PLA)	Anaerobic digestion	Synthesized kitchen waste (Cabbage, Potato, Apple, Carrot , Fish, Pork (raw) , Rice, Tea leaves, Used paper,	1 L	52 °C	8 weeks	Laboratory	PLA (50%): 37%<br><br>PLA (90%) 80%<br><br>PLA (80%): 52%	PLA (50%): 276 NL/kg-VS<br><br>PLA (90%): 598 NL/kg-VS<br><br>PLA (80%): 389 NL/kg-VS
[42]	Poly (lactic acid) (PLA)	Anaerobic digestion	-	1.5 L	37 and 55 °C	75 days	Laboratory	PLA (35 ºC): 21%<br>PLA (55ºC): 93%	PCL: 10.6 L<br>PLA: 6.86
[43]	Poly (lactic acid) (PLA)<br>Poly caprolactone (PCL)	Anaerobic digestion	-	1.5 L	55 °C	75 days	Laboratory	PCL: 92%<br>PLA: 79%	PCL: 10.6 L<br>PLA: 6.86
[44]	Poly lactic acid (PLA)	Landfill	Municipal solid waste	-	-	15 months	real conditions	-	-
[45]	Degradable plastic additive 3%<br>Biodegradable synthetic resin	Anaerobic digestion	-	1 L	-	32 days	Laboratory	Plastic with additive: 0.02<br><br>Biodegradable synthetic resin: 26.9	245 ml MB
[46]	Polyurethane (PU)	Anaerobic digestion	-	1 L	-	45 days	Laboratory	0	-
[47]	Poly (vinyl chloride) (PVC)	Landfill	Municipal solid waste	Real	35 °C	90 days	Real conditions	-	-
[48]	Poly (vinyl chloride) (PVC)	Landfill	Artificial municipal solid waste	5 L	20, 37, 55 and 70 °C	40 days	Laboratory	-	-
[49]	Polycaprolactone (PCL)<br>Polylactic acid (PLA)<br>Blend starch C polycaprolactone<br>Poly (butadiene adipate-co-terephthalate)	Anaerobic digestion	-	250 mL	35 °C	28 days	Laboratory	-	-
[50]	polyhydroxybutyrate (PHB)<br>(hydroxybutyrate-*co*-hydroxyvalerate) (PHBV)<br>Poly (ε-caroplacton) (PCL)	Anaerobic digestion	-	-	37 °C	42 days	Laboratory	PHB: 100<br>PHBV: 57<br>PCL: 30	-

Reference	Type plastic	Degradation	Co-sustrate	Working volume	Temperature	Duration	Scale	% Biodegradation	Biogas
[51]	polyhydroxybutyrate and hydroxyvalerate (PHBV) polycaprolactone plastic (PCL) Blend of starch and polyvinyl alcohol (SPVA) ellulose acetate (CA)	Anaerobic digestion<br><br>Landfill	Synthesized waste which contained equivalent weights of yard waste, kitchen garbage, paper waste, dry dog-food, dewatered sludge and soil	300 mL	-	120 days	Laboratory	PHBV: 100%	-
[52]	Poly(3-hydroxybutyrate-*co*-3-hydroxyoctanoate), PHBO C-PCL (poly-ε-caprolactone)	Anaerobic digestion<br><br>Landfill	Mixture of shredded fresh MSW	500mL	35 °C<br><br>38 °C	721 days	Laboratory	PHBO: 45.2<br><br>C-PCL: 40	-
[53]	Polyhydroxybutyrate and hydroxyvalerate (PHBV) Polyhydroxybutyrate (PHB)	Anaerobic digestion	-	1 L	37 °C	-	Laboratory	-	-
[54]	Poly (vinyl chlor- ide) PVC	Landfill	Household waste	100 L	30 °C	4 years	Laboratory	-	-
[55]	Poly (vinyl chloride) (PVC)	Landfill	Household waste	100 L	30 °C	-	Laboratory	-	-
[56]	low-density polyethylene film (LDPE)	Landfill	-	-	25, 50, 80 and 105 °C	-	Laboratory	-	-
[57]	low-density polyethylene film (LDPE) /rice starch	Landfill	-	-	37 °C	180 days	Laboratory	LDPE/rice starch (60/40): 21.4	-
[58]	Lignified cellulose (LC), a citric acid cross-linked cellulose (x-C),	Landfill	Fresh refuse, and decompod refuse	2 L	38 °C	6 months	Laboratory	LC: 25.67<br>XC: 52.04	-
[59]	low-density polyethylene film (LDPE) Starch	Landfill	-	-	37 °C	180 days	Laboratory	LDPE/starch (60/40): 27.3	-
[60]	Poly-3-hydroxybutyrate-co-3-hydroxyvalerate (PHBV)	Anaerobic digestion	-	500 mL	35 °C	30 days	Laboratory	90%	-
[61]	Poly (3-hydroxy- butyrate-co-3-hydroxyvalerate) (PHB/HV; 92/8, w/w)<br>Poly-lactic acid	Anaerobic digestion<br><br>Landfill	Synthetic MSW	160 mL	35 °C	100 days<br><br>6 months	Laboratory	89%<br><br>PHB/HV: 80%	-
[62]	Starch acetates	Anaerobic digestion	Municipal solid waste	3.5 L	37 °C	90 days	Laboratory	-	-
[63]	Poly-β-hydroxyalkanoates	Anaerobic digestion	-	125 mL	15 °C	-	Laboratory	-	-
[64]	Polylactic acid<br>Polylactone, and poly(hydroxy butyrate/valerate)<br>Starch/PE	Anaerobic digestion	-	160 mL	35 °C	40 days	Laboratory	-	-

Reference	Type plastic	Degradation	Co-sustrate	Working volume	Temperature	Duration	Scale	% Biodegradation	Biogas
[65]	Poly-ε-caprolactone	Anaerobic digestion	-	-	30 °C	7 days	Laboratory	-	-
[66]	Low-density polyethylene film (LDPE) starch-based/between 70 and 90% starch	Anaerobic digestion	--	119 mL	30 °C	28 days	Laboratory	-	-
[67]	Low-density polyethylene (LDPE)<br>LDPE + 5.5% cornstarch<br>Linear low-density polyethylene (LLDPE)<br>LLDPE + 5.5% cornstarch	Landfill	Municipal solid waste	-	10 °C	2 years	Real conditions	-	-
[68]	Poly (3-hydroxybutyrate)<br>Copolymerpoly(3-hydroxybutyrate-co-3-hydroxyvalerate)	Anaerobic digestion	-	59 mL	35 °C	16 days	Laboratory	PHB: 87<br>P (HB-co-13%HV): 96<br>P (HB-co-20%HV): 83	PHB: 0.20 mmol<br>P(HB-co-13%HV): 0.23 mmol<br>P(HB-co-20%HV): 0.20 mmol
[69]	Copolymer of cellulose acetate<br>Polystyrene maleic anhydride (50/50%)	Anaerobic digestion	-	155 mL	37 °C	98 days	Laboratory	-	-
[70]	Polyethylene (PE) + 6% starch (A; B, and C)<br><br>PE +10-12% starch<br>PHB/PHV<br>PVA	Anaerobic digestion	-	-	58 °C	60 days	Laboratory	PHB/PHV: 91.4	-

References

1. Zhang, W.; Heaven, S.; Banks, C.J. Degradation of some EN13432 compliant plastics in simulated mesophilic anaerobic digestion of food waste. *Polym. Degrad. Stab.* **2018**, *147*, 76–88.
2. Wang, S.; Lydon, K.A.; White, E.M.; Grubbs III, J.B.; Lipp, E.K.; Locklin, J.; Jambeck, J.R. Biodegradation of Poly(3-hydroxybutyrate-co-3-hydroxyhexanoate) Plastic under Anaerobic Sludge and Aerobic Seawater Conditions: Gas Evolution and Microbial Diversity. *Environ. Sci. Technol.* **2018**, *52*, 5700–5709.
3. Lim, J.W.; Ting, D.W.Q.; Loh, K.-C.; Ge, T.; Tong, Y.W. Effects of disposable plastics and wooden chopsticks on the anaerobic digestion of food waste. *Waste Manag.* **2018**, *79*, 607–614.
4. Ryan, C.A.; Billington, S.L.; Criddle, C.S. Biocomposite fiber-matrix treatments that enhance in-service performance can also accelerate end-of-life fragmentation and anaerobic biodegradation to methane. *J. Poly. Environ.* **2018**, *26*, 1715–1726.
5. Xia, T.; Huang, H.; Wu, G.; Sun, E.; Jin, X.; Tang, W. The characteristic changes of rice straw fibers in anaerobic digestion and its effect on rice straw-reinforced composites. *Ind. Crop. Prod.* **2018**, *121*, 73–79.
6. Ryan, C.A.; Billington, S.L.; Criddle, C.S. Methodology to assess end-of-life anaerobic biodegradation kinetics and methane production potential for composite materials. *Compos. Part A: Appl. Sci. Manuf.* **2017**, *95*, 388–399.
7. Ryan, C.A.; Billington, S.L.; Criddle, C.S. Assessment of models for anaerobic biodegradation of a model bioplastic: Poly(hydroxybutyrate-co-hydroxyvalerate). *Bioresour. Technol.* **2017**, *227*, 205–213.
8. Moon, J.; Kim, M.Y.; Kim, B.M.; Lee, J.C.; Choi, M.-C.; Kim, J.R. Estimation of the Microbial Degradation of Biodegradable Polymer, Poly(lactic acid) (PLA) with a Specific Gas Production Rate. *Macromol. Res.* **2016**, *24*, 415–421.
9. Boonmee, J.; Kositanont, C.; Leejarkpa, T. Biodegradation of poly (lactic acid), poly (hydroxybutyrate-co-hydroxyvalerate), poly (butylene succinate) and poly (butylene adipate-co-terephthalate) under anaerobic and oxygen limited thermophilic conditions. *EnvironmentAsia* **2016**, 9.
10. Adamcova, D.; Vaverková, M.D. New Polymer Behavior Under the Landfill Conditions. *Waste Biomass-Valorization* **2016**, *7*, 1459–1467.
11. Šmejkalová, P.; Kužníková, V.; Merna, J.; Hermanová, S.; Mejkalova, P.; Nikova, V.K. Anaerobic digestion of aliphatic polyesters. *Water Sci. Technol.* **2016**, *73*, 2386–2393.
12. Liu, W.W.; Xue, J.; Cheng, B.J.; Zhu, S.W.; Ma, Q.; Ma, H. Anaerobic biodegradation, physical and structural properties of normal and high-amylose maize starch films. *Int. J. Agric. & Biol. Eng.* **2016**, *9*, 184–193.
13. Lee, J.C.; Moon, J.H.; Jeong, J.H.; Kim, M.Y.; Kim, B.M.; Choi, M.C.; Ha, C.S. Biodegradability of poly (lactic acid)(PLA)/lactic acid (LA) blends using anaerobic digester sludge. *Macromol. Res.* **2016**, *24*, 741–747.
14. Nauendorf, A.; Krause, S.; Bigalke, N.K.; Gorb, E.V.; Gorb, S.N.; Haeckel, M.; Treude, T. Microbial colonization and degradation of polyethylene and biodegradable plastic bags in temperate fine-grained organic-rich marine sediments. *Mar. Pollut. Bull.* **2016**, *103*, 168–178..
15. Mackuľak, T.; Takáčová, A.; Gál, M.; Marton, M.; Ryba, J. PVC degradation by Fenton reaction and biological decomposition. *Polym. Degrad. Stab.* **2015**, *120*, 226–231.
16. Dvořáčková, M.; Svoboda, P.; Kostka, L.; Pekařová, S. Influence of biodegradation in thermophilic anaerobic aqueous conditions on crystallization of poly(butylene succinate). *Polym. Test.* **2015**, *47*, 59–70.
17. Petit, M.G.; Correa, Z.; Sabino, M.A. Degradation of a Polycaprolactone/Eggshell Biocomposite in a Bioreactor. *J. Poly. Environ.* **2015**, *23*, 11–20.
18. Muenmee, S.; Chiemchaisri, W.; Chiemchaisri, C. Microbial consortium involving biological methane oxidation in relation to the biodegradation of waste plastics in a solid waste disposal open dump site. *Int. Biodeterior. Biodegradation* **2015**, *102*, 172–181.
19. Iwańczuk, A.; Kozłowski, M.; Łukaszewicz, M.; Jabłoński, S. Anaerobic Biodegradation of Polymer Composites Filled with Natural Fibers. *J. Polym. Environ.* **2015**, *23*, 277–282.
20. Selke, S.; Auras, R.; Nguyen, T.A.; Castro Aguirre, E.; Cheruvathur, R.; Liu, Y. Evaluation of biodegradation-promoting additives for plastics. *Environ. Sci. Technol.* **2015**, *49*, 3769–3777.
21. Hermanová, S.; Šmejkalová, P.; Merna, J.; Zarevúcka, M. Biodegradation of waste PET based copolyesters in thermophilic anaerobic sludge. *Polym. Degrad. Stab.* **2015**, *111*, 176–184.
22. Adamcová, D.; Vaverková, M. Degradation of biodegradable/degradable plastics in municipal solid-waste landfill. *Polish J. Environ. Stud.* **2014**, *23*, 1071–1078.

23. Gómez, E.F.; Luo, X.; Li, C.;. Michel, F.C; Li, Y. Biodegradability of crude glycerol-based polyurethane foams during composting, anaerobic digestion and soil incubation. *Polym. Degrad. Stab.* **2014**, *102*, 195–203.
24. Masood, F.; Yasin, T.; Hameed, A. Comparative oxo-biodegradation study of poly-3-hydroxybutyrate-co-3-hydroxyvalerate/polypropylene blend in controlled environments. *Int. Biodeterior. Biodegradation* **2014**, *87*, 1–8.
25. Yagi, H.; Ninomiya, F.; Funabashi, M.; Kunioka, M. Mesophilic anaerobic biodegradation test and analysis of eubacteria and archaea involved in anaerobic biodegradation of four specified biodegradable polyesters. *Polym. Degrad. Stab.* **2014**, *110*, 278–283.
26. Gómez, E.F.; Michel, F.C. Biodegradability of conventional and bio-based plastics and natural fiber composites during composting, anaerobic digestion and long-term soil incubation. *Polym. Degrad. Stab.* **2013**, *98*, 2583–2591.
27. Yagi, H.; Ninomiya, F.; Funabashi, M.; Kunioka, M. Thermophilic anaerobic biodegradation test and analysis of eubacteria involved in anaerobic biodegradation of four specified biodegradable polyesters. *Polym. Degrad. Stab.* **2013**, *98*, 1182–1187.
28. Kolstad, J.J.; Vink, E.T.H.; De Wilde, B.; Debeer, L. Assessment of anaerobic degradation of Ingeo™ polylactides under accelerated landfill conditions. *Polym. Degrad. Stab.* 201297, 1131–1141.
29. Yagi, H.; Ninomiya, F.; Funabashi, M.; Kunioka, M. Anaerobic Biodegradation of Poly (Lactic Acid) Film in Anaerobic Sludge. *J. Polym. Environ.* **2012**, *20*, 673–680.
30. Tollner, E.W.; Annis, P.A.; Das, K.C. Evaluation of Strength Properties of Polypropylene-Based Polymers in Simulated Landfill and Oven Conditions. *J. Environ. Eng.* **2011**, *137*, 291–296.
31. Cho, H.S.; Moon, H.S.; Kim, M.; Nam, K.; Kim, J.Y. Biodegradability and biodegradation rate of poly(caprolactone)-starch blend and poly(butylene succinate) biodegradable polymer under aerobic and anaerobic environment. *Waste Manag.* **2011**, *31*, 475–480.
32. Yagi, H.; Ninomiya, F.; Funabashi, M.; Kunioka, M. RNA analysis of anaerobic sludge during anaerobic biodegradation of cellulose and poly(lactic acid) by RT-PCR–DGGE. *Polym. Degrad. Stab.* **2011**, *96*, 547–552.
33. Morse, M.C.; Liao, Q.; Criddle, C.S.; Frank, C.W. Anaerobic biodegradation of the microbial copolymer poly(3-hydroxybutyrate- co-3-hydroxyhexanoate): Effects of comonomer content, processing history, and semi-crystalline morphology. *Polymer (Guildf).* **2011**, *52*, 547–556.
34. Guo, M.; Trzcinski, A.P.; Stuckey, D.C.; Murphy, R.J. Anaerobic digestion of starch–polyvinyl alcohol biopolymer packaging: Biodegradability and environmental impact assessment. *Bioresour. Technol.* **2011**, *102*, 11137–11146.
35. Wang, F.; Tsuno, H.; Hidaka, T.; Tsubota, J. Promotion of polylactide degradation by ammonia under hyperthermophilic anaerobic conditions. *Bioresour. Technol.* **2011**, *102*, 9933–9941.
36. Shi, B.; Palfery, D. Enhanced mineralization of PLA meltblown materials due to plasticization. *J. Polym. Environ.* **2010**, *18*, 122–127.
37. Yagi, H.; Ninomiya, F.; Funabashi, M.; Kunioka, M. Bioplastic biodegradation activity of anaerobic sludge prepared by preincubation at 55°C for new anaerobic biodegradation test. *Polym. Degrad. Stab.* **2010**, *95*, 1349–1355.
38. Vargas, L.F.; Welt, B.A.; Teixeira, A.; Pullammanappallil, P.; Balaban, M.; Beatty, C. Biodegradation of treated polylactic acid (PLA) under anaerobic conditions. *Trans. ASABE* **2009**, *52*, 1025–1030.
39. Gutierrez-Wing, M.T.; Stevens, B.E.; Theegala, C.S.; Negulescu, I.I.; Rusch, K.A.; M.ASCE, P.E. Anaerobic biodegradation of polyhydroxybutyrate in municipal sewage sludge. *J. Environ. Eng.* **2010**, *136*, 709–718.
40. Russo, M.A.L.; O'Sullivan, C.; Rounsefell, B.; Halley, P.J.; Truss, R.; Clarke, W.P. The anaerobic degradability of thermoplastic starch: Polyvinyl alcohol blends: Potential biodegradable food packaging materials. *Bioresour. Technol.* **2009**, *100*, 1705–1710.
41. Xiao, D.; Matsuda, J.; Liu, B.; Ohmiya, K. Characteristics of fermentation of biodegradable plastics mixed with household solid waste by thermophilic dry anaerobic co-digestion. *J. Japanese Soc. Agric. Mach.* **2009**, *71*, 55–62.
42. Yagi, H.; Ninomiya, F.; Funabashi, M.; Kunioka, M. Anaerobic biodegradation tests of poly(lactic acid) under mesophilic and thermophilic conditions using a new evaluation system for methane fermentation in anaerobic sludge. *International J. Mol. Sci.* **2009**, *10*, 3824–3835.
43. Yagi, H.; Ninomiya, F.; Funabashi, M.; Kunioka, M. Anaerobic biodegradation tests of poly(lactic acid) and polycaprolactone using new evaluation system for methane fermentation in anaerobic sludge. *Polym. Degrad. Stab.* **2009**, *94*, 1397–1404.

44. Rudeekit, Y.; Numnoi, J.; Tajan, M.; Chaiwutthinan, P.; Leejarkpai, T. Determining biodegradability of polylactic acid under different environments. *J. Met. Mater. Miner.* **2008**, *18*, 83–87.
45. Mohee, R.; Unmar, G.D.; Mudhoo, A.; Khadoo, P. Biodegradability of biodegradable/degradable plastic materials under aerobic and anaerobic conditions. *Waste Manag.* **2008**, *28*, 1624–1629.
46. Urgun-Demirtas, M.; Singh, D.; Pagilla, K. Laboratory investigation of biodegradability of a polyurethane foam under anaerobic conditions. *Polym. Degrad. Stab.* **2007**, *92*, 1599–1610.
47. Grossman, R.F.; Schleicher Jr., J.E.; D'Alessio, L. Considerations affecting landfill biodegradability of PVC. *J. Vinyl Addit. Technol.* **2007**, *13*, 133–135.
48. Björn, A.; Hörsing, M.; Karlsson, A.; Mersiowsky, I.; Ejlertsson, J. Impacts of temperature on the leaching of organotin compounds from poly(vinyl chloride) plastics-A study conducted under simulated landfill conditions. *J. Vinyl Addit. Technol.* **2007**, *13*, 176–188.
49. Massardier-Nageotte, V.; Pestre, C.; Cruard-Pradet, T.; Bayard, R. Aerobic and anaerobic biodegradability of polymer films and physico-chemical characterization. *Polym. Degrad. Stab.* **2006**, *91*, 620–627.
50. Abou-Zeid, D.; Müller, R.-J.; Deckwer, W.-D. Biodegradation of aliphatic homopolyesters and aliphatic−aromatic copolyesters by anaerobic microorganisms. *Biomacromolecules* **2004**, *5*, 1687–1697, 2004.
51. Ishigaki, T.; Sugano, W.; Nakanishi, A.; Tateda, M.; Ike, M.; Fujita, M. The degradability of biodegradable plastics in aerobic and anaerobic waste landfill model reactors. *Chemosphere*, **2004**, *54*, 225–233.
52. Federle, T.W.; Barlaz, M.A.; Pettigrew, C.A.; Kerr, K.M.; Kemper, J.J.; Nuck, B.A.; Schechtman, L.A. Anaerobic biodegradation of aliphatic polyesters: poly(3-hydroxybutyrate- co -3-hydroxyoctanoate) and poly(ε-caprolactone). *Biomacromolecules* **2002**, *3*, 813–822.
53. Abou-Zeid, D.-M.; Müller, R.-J.; Deckwer, W.-D. Degradation of natural and syntehtic polyesters under anaerobic conditions. *J. Biotechnol.* **2001**, *86*, 113–126.
54. Mersiowsky, I.; Weller, M.; Ejlertsson, J. Fate of plasticised PVC products under landfill conditions: a laboratory-scale landfill simulation reactor study. *Water Res.* **2001**, *35*, 3063–3070.
55. Mersiowsky, I.; Stegmann, R.; Ejlertsson, J. Long term behaviour of poly(vinyl chloride) products under soil buried and landfill conditions. *Plast. Rubber Compos.* **1999**, *28*, 321–326.
56. Haider, N.; Karlsson, S. Migration and release profile of Chimassorb 944 from low-density polyethylene film (LDPE) in simulated landfills. *Polym. Degrad. Stab.* **1999**, *64*, 321–328.
57. Arvanitoyannis, I.; Biliaderis, C.G.; Ogawa, H.; Kawasaki, N. Biodegradable films made from low-density polyethylene (LDPE), rice starch and potato starch for food packaging applications: Part 1. *Carbohydr. Polym.* **1998**, *36*, 89–104.
58. Ress, B.B.; Calvert, P.P.; Pettigrew, C.A.; Barlaz, M.A. Testing anaerobic biodegradability of polymers in a laboratory-scale simulated landfill. *Environ. Sci. Technol.*, **1998**, *32*, 821–827.
59. Psomiadou, E.; Arvanitoyannis, I.; Biliaderis, C.G.; Ogawa, H.; Kawasaki, N. Biodegradable films made from low density polyethylene (LDPE), wheat starch and soluble starch for food packaging applications. Part 2. *Carbohydr. Polym.* **1997**, *33*, 227–242.
60. Reischwitz, A.; Stoppok, E.; Buchholz, K. Anaerobic degradation of poly-3-hydroxybutyrate and poly-3-hydroxybutyrate-co-3-hydroxyvalerate. *Biodegradation* **1997**, *8*, 313–319.
61. Shin, P.K.; Kim, M.H.; Kim, J.M. Biodegradability of degradable plastics exposed to anaerobic digested sludge and simulated landfill conditions. *J. Environ. Polym. Degrad.* **1997**, *5*, 33–39.
62. Rivard, C.; Moens, L.; Roberts, K.; Brigham, J.; Kelley, S. Starch esters as biodegradable plastics: Effects of ester group chain length and degree of substitution on anaerobic biodegradation. *Enzyme Microb. Technol.* **1995**, *17*, 848–852.
63. Mas-Castellà, J.; Urmeneta, J.; Lafuente, R.; Navarrete, A.; Guerrero, R. Biodegradation of Poly-β-hydroxyalkanoates in anaerobic sediments. *Int. Biodeterior. Biodegradation* **1995**, *35*, 155–174.
64. Day, M.; Shaw, K. ; Cooney, D. Biodegradability: An assessment of commercial polymers according to the Canadian method for anaerobic conditions. *J. Environ. Polym. Degrad.* **1994**, *2*, 121–127.
65. Nishida, H.; Tokiwa, Y. Confirmation of anaerobic poly(2-oxepanone) degrading microorganisms in environments. *Chem. Lett.* **1994**, *23*, 1293–1296.
66. van der Zee, M.; Sijtsma, L.; Tan, G.B.; Tournois, H.; de Wit, D. Assessment of biodegradation of water insoluble polymeric materials in aerobic and anaerobic aquatic environments. *Chemosphere* **1994**, *28*, 1757–1771.
67. Breslin, V.T. Degradation of starch-plastic composites in a municipal solid waste landfill. *J. Environ. Polym. Degrad.* **1993**, *1*, 127–141.

68. Budwill, K.; Fedorak, P.M.; Page, W.J. Methanogenic degradation of poly(3-hydroxyalkanoates). *Appl. Environ. Microbiol.* **1992**, *58*, 1398–1401.
69. Rivard, C.J.; Adney, W.S.; Himmel, M.E.; Mitchell, D.J.; Vinzant, T.B.; Grohmann, K.; Chum, H. Effects of natural polymer acetylation on the anaerobic bioconversion to methane and carbon dioxide. *Appl. Biochem. Biotechnol.* **1992**, *34*, 725–736.
70. Krupp, L.R.; Jewell, W.J. Biodegradability of modified plastic films in controlled biological environments. *Environ. Sci. Technol.* **1992**, *26*, 193–198.

© 2019 by the authors. Submitted for possible open access publication under the terms and conditions of the Creative Commons Attribution (CC BY) license (http://creativecommons.org/licenses/by/4.0/).
